# Agrin‐Enriched Neonatal Spinal Cord‐Derived Extracellular Vesicles Bypass Age Barriers to Organize Spinal Cord Repair

**DOI:** 10.1002/jev2.70371

**Published:** 2026-10-10

**Authors:** Yongfeng Zhang, Qi Liu, Beibei Yu, Borui Xue, Shijie Yang, Teng Ma, Lingli Guo, Haining Wu, Xue Gao, Shengyou Li, Yujie Yang, Yiming Hao, Ziqing Zhu, Meijia Su, Hongyu Li, Shuanshuan Fan, Jiaqi Wang, Huangtao Chen, Longhui Fu, Anhui Qin, Zhenguo Wang, Shihao Nie, Mingze Qin, Xinxin Bai, Guang Kong, Rui Zhao, Dong Lei, Yue Yu, Junxiong Ma, Shouping Gong, Bing Xia, Jinghui Huang

**Affiliations:** ^1^ Department of Orthopaedics Xijing Hospital, Fourth Military Medical University Xi'an People's Republic of China; ^2^ Department of Neurology The First Affiliated Hospital of Xi'an Jiao Tong University Xi'an People's Republic of China; ^3^ Department of Neurosurgery The First Affiliated Hospital of Chongqing Medical University Chongqing People's Republic of China; ^4^ 986th Hospital of Air Force Fourth Military Medical University Xi'an People's Republic of China; ^5^ Department of Orthopaedics and Traumatology, School of Clinical Medicine the University of Hong Kong Hong Kong People's Republic of China; ^6^ Military Medical Innovation Center Fourth Military Medical University Xi'an People's Republic of China; ^7^ College of Basic Medicine Shaanxi University of Chinese Medicine Xianyang People's Republic of China; ^8^ Department of Neurosurgery The Second Affiliated Hospital of Xi'an Jiao Tong University Xi'an People's Republic of China; ^9^ Department of Orthopedics General Hospital of Northern Theater Command Shenyang People's Republic of China; ^10^ Xi'an Medical University Xi'an People's Republic of China

## Abstract

Spinal cord injury (SCI) poses a formidable challenge in regenerative medicine, with current therapeutic strategies proving largely unsuccessful in achieving functional repair. Notably, paediatric patients and neonatal mice exhibit spontaneous and efficient regeneration after SCI, while the mechanisms remain elusive. Through integrated multi‐omics analysis in neonatal, adult and aged mice with SCI, we discovered that the regeneration time window, inflammatory response, and intercellular communication are all age‐dependent. Neonatal spinal cord‐derived extracellular vesicles (nSC‐EVs) were identified carrying a regenerative protein signature and were critical mediators in restoring SCI repair in neonatal mice. Functionally, nSC‐EVs promote neurogenesis *via* the Wnt/β‐catenin pathway and mediate axonal growth through the core effector Agrin (encoded by *Agrn*), which was found to be predominantly expressed in endothelial cells. We further developed a targeted delivery system for nSC‐EVs, which significantly enhanced functional recovery, including motor and bladder control, in adult SCI mice. This work elucidates the mechanistic basis of age‐dependent repair processes and proposes a novel EV‐based therapy for SCI.

## 1 Introduction

Spinal cord injury (SCI) remains a devastating neurological disorder characterized by irreversible sensorimotor deficits. Annually, there are over 90 new cases of SCI per million people globally (GBD Spinal Cord Injuries Collaborators, 2023). While experimental therapies targeting neuroprotection and plasticity have advanced, clinical translation remains impeded by the limited regenerative capacity of the adult mammalian central nervous system (CNS). Strikingly, pediatric patients retain partial repair capabilities after SCI, whereas elderly individuals exhibit universally impaired recovery (Parent et al. 2011; Kirshblum et al.^2021^; Canton et al. 2019; Kvistad et al. 2024). This dichotomy is mirrored in rodents: neonatal mice achieve scar‐free regeneration with functional restoration, while adult or aged mice develop gliotic scars and fail to reconstitute neural circuitry (Li et al. 2020; Salvador et al. 2023; Skinnider et al. 2024). This age‐dependent disparity underscores the critical significance of understanding endogenous repair mechanisms, particularly the temporal regulation of molecular and cellular effectors in the spinal cord.

Emerging evidence increasingly supports the view that age‐related declines in tissue repair capacity are not solely attributed to cell‐intrinsic deficits but also reflect systemic imbalances in multicellular coordination (Cai et al. 2023; Ji et al. 2023; López‐Otín et al. 2013). As the aging process advances, intercellular communication networks progressively deteriorate, introducing pathogenic noise that disrupts homeostatic regulation and tissue repair competence (Baruch et al. 2014; López‐Otín et al. 2023). The erosion of intercellular communication particularly undermines critical growth factor signalling cascades essential for tissue regeneration (Li et al. 2024, 2025; Su et al. 2024). Previous studies using heterochronic parabiosis experiments with young and old mice demonstrated that exposure to a young systemic environment partially restores physical and immune fitness in aged parabionts, which indicated the critical influence of the systemic environment on the rejuvenation of aged tissues and thus underscores the regulatory role of altered intercellular communication during aging in tissue regenerative potential (Brigger et al. 2020; Conboy et al. 2005). Consequently, systematic deconstruction of these cellular interaction networks and restore communicative fidelity in aged tissues may represent an essential frontier for deciphering the principles of age‐impaired regeneration.

Extracellular vesicles (EVs) are crucial mediators of intercellular signalling that convey parent cell‐derived information, exhibiting distinct age‐specific signatures (Kisielewska et al. 2024; Luo et al. 2023; Salomon et al. 2022; Urbanelli et al. 2016). However, current research on EVs predominantly focuses on monoculture systems of isolated cells *in vitro* (Fuhrmann et al. 2015). These culture conditions fail to recapitulate the spatiotemporal complexity of the physiological cellular milieu, potentially suppressing intrinsic cellular functions or inducing adverse effects (Kusuma et al. 2022; Urzì et al. 2022). Consequently, EVs derived from single‐source cultured cells are produced by cells adapted to artificial monolayer culture conditions, which often results in genetic perturbations and loss of tissue‐specific functions (Almeria et al. 2024; Jalilian et al. 2022). Over recent years, emerging evidence has demonstrated that tissue‐derived EVs exhibit significant advantages over their cell‐derived counterparts in recapitulating *in vivo* pathophysiology and therapeutic potential (Li et al. 2025; Ying et al. 2024). Critically, tissue‐derived EVs originate from cells within their native tissue microenvironment, preserving complex intercellular communication networks, physiological stressors and tissue‐specific biomolecular cargo (e.g., miRNAs, proteins, lipids) shaped by authentic cell‐cell and cell‐matrix interactions (Lou et al. 2023; van Niel et al. 2022). Therefore, tissue‐derived EVs more accurately reflect the heterogeneous EV population present *in vivo*, carrying tissue‐specific spatiotemporal signatures absent in cell‐derived EVs.

Recent advances in tissue‐derived EVs research have overcome limitations of previous *in vitro* cell‐cultured EVs, such as restricted sources and compositionally limited profiles, enablling EVs to play unique and potent roles in tissue repair across various organ systems, including liver, cardiac, bone, skin, and renal repair (An et al. 2021; Li et al. 2025; Li and Yu 2024; Lou et al. 2023; Yang et al. 2023). However, CNS regeneration is a complex process heavily influenced by the regenerative niche, and there are currently few studies reporting the injury repair effects of tissue‐derived EVs. Thus, deciphering the age‐dependent cellular communication and molecular landscapes of the spinal cord is crucial for bridging knowledge gaps. Furthermore, elucidating the role of tissue‐derived EVs, which are capable of closely recapitulating niche signalling, in neural repair processes may offer novel insights into SCI repair.

Herein, we demonstrate that spinal cord tissue‐derived EVs (SC‐EVs) serve as pivotal mediators in coordinating the repair process after SCI and conveying functionally active molecules that underlie the neonatal‐specific robust neural regenerative capacity. These findings provide novel mechanistic insights into age‐related disparity of repair competence and suggest promising therapeutic strategies for rejuvenating regenerative potential. Furthermore, we found that SC‐EVs from neonatal mice (nSC‐EVs) markedly promoted the proliferation of neural stem cells (NSCs) by activating the Wnt/β‐catenin pathway, and mediated axonal growth through the endothelial signalling Agrin (*Agrn*). Further, building on previous studies (Shen et al. 2021; Yang et al. 2025), we functionalized nSC‐EVs with activatable cell‐penetrating peptides (ACPPs) to target the injury site, which significantly promoted SCI repair in adult mice. This study establishes nSC‐EVs as age‐specific modulators of spinal cord regenerative capacity and demonstrates that harnessing nSC‐EVs‐mediated intercellular communication offers a rejuvenation strategy to restore adult nerve regenerative potential following SCI.

## 2 Results

### 2.1 Phenotypic and Molecular Disparity of SCI Repair in Neonatal, Adult and Aged Mice

To elucidate age‐dependent mechanisms of SCI repair, we conducted comprehensive analyses across neonatal (postnatal day 2, P2), young adult (8–10 weeks old, 8–10 w), and aged (24 months old, 24 m) mice following crush injury (Figure S1A and S1B). Our integrated approach combined longitudinal functional assessment, histological characterization and multi‐omics profiling to establish a mechanistic framework for developmental stage‐specific repair capacities (Figure 1A and 1B).

**FIGURE 1:**
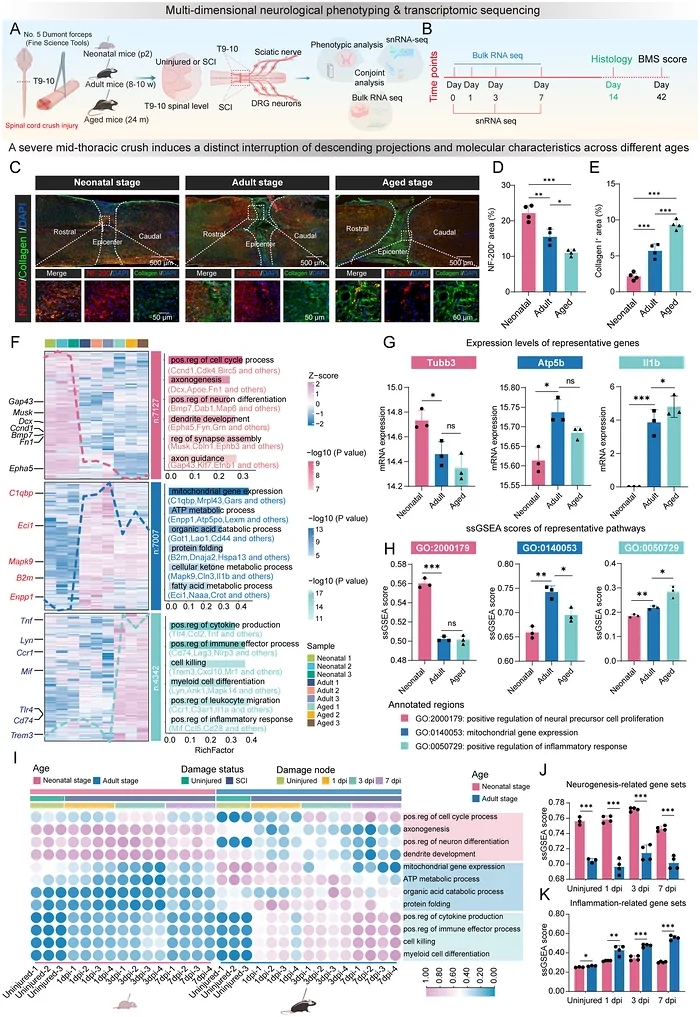
**Differences in SCI repair in neonatal, adult and aged mice and the molecular mechanisms**. (**A**) Flowchart of the experimental design. (**B**) The time points for the observation of bulk RNA sequencing, snRNA‐seq, histological, and behavioural outcome measures. (**C**) Representative immunofluorescence images at 14 dpi in neonatal, adult, and aged mice following SCI, showing the distribution of NF‐200^+^ and Collagen I^+^ in the SCI regions across different age groups. (**D**–**E**) Quantitative analysis of the NF‐200^+^ cell area (D) and Collagen I^+^ cell area (E) (*n* = 4). (**F**) The heatmap (left) depicts gene expression patterns in the spinal cord across age groups, while the bar graph (right) presents the statistical significance of enriched biological processes for each functional cluster. Bar length represents the rich factor, and color intensity indicates statistical significance (–log_10_ *P* value). (**G**) The expression differences of three representative genes were specifically analyzed across age groups: the neuronal development marker *Gap43*, the mitochondrial function gene *Atp5b*, and the pro‐inflammatory gene *Il1b* (*n* = 3). (**H**) Quantitative analysis of ssGSEA scores for differentially enriched pathways across age groups (*n* = 3). (**I**) Dynamic changes of high‐activity functional gene sets of different age groups in neonatal versus adult mouse spinal cords pre‐ and post‐injury (1, 3, and 7 dpi). (**J** to **K**) ssGSEA scores of gene sets associated with neural regeneration (J) and inflammation/cell death (K) post‐SCI in neonatal and adult mice. Statistical significance was determined by one‐way ANOVA followed by Tukey's multiple comparisons post hoc test (D, E, G and H) or t‐test (J and K). Data in D, E, G, H, J and K are presented as mean ± SD. Significance levels are indicated as ns (not significant, *P* ≥ 0.05), ^*^ *P* < 0.05, ^**^ *P* < 0.01, and ^***^ *P* < 0.001.

Histological evaluation at 14 days post injury (dpi) revealed an inverse relationship between regenerative potential and fibrotic scarring across age groups. The area of NF‐200^+^ regions was the largest in neonatal mice, intermediate in adult mice, and smallest in aged mice (Figure 1C and D). Conversely, the area of Collagen I^+^ cells, a marker of scar formation, exhibited an opposing trend: it was smallest in neonatal mice, intermediate in adult mice, and largest in aged mice (Figure 1C and E). These findings suggest that the capacity for tissue regeneration following SCI declines with increasing age, while scar formation is enhanced. These structural differences correlated with functional outcomes, where neonatal mice achieved significantly better motor recovery compared to adult mice and aged mice examined by Basso Mouse Scale (BMS) scoring at 42 dpi (Figure S1C).

Transcriptomic profiling systematically uncovered molecular expression disparities in spinal cord tissues across distinct age groups of mice. Mfuzz cluster analysis identified 7127 neonatal‐enriched genes associated with cell cycle regulation and axonogenesis, contrasting with 7007 adult‐specific genes involved in mitochondrial metabolism and 4342 aged‐associated genes related to inflammatory regulation and cell killing signalling (Figure 1F, Data S1). To further validate these findings, we analyzed the differential expression patterns of representative key genes and pathways enriched in each cluster. Results showed that the neuronal development marker *Gap43* exhibited peak expression during the neonatal stage, significantly higher than in adult and aged stages. Meanwhile, the mitochondrial function gene *Atp5b* displayed an age‐dependent trend, with maximal expression in the adult group. In contrast, the inflammatory factor *Il1b* was significantly upregulated with advancing age (Figure 1G). Functional module analysis corroborated these trends. Single‐sample GSEA (ssGSEA) quantification confirmed this developmental continuum, with positive regulation of neural progenitor proliferation (GO:2000179) dominating in neonates, mitochondrial gene expression (GO:0140053) in adults, and positive regulation of inflammatory responses (GO:0050729) in aged mice (Figure 1H, Data S2).

This section concludes that neonatal mice exhibit superior recovery after SCI compared to adult and aged mice. Bioinformatics approaches elucidated the molecular mechanisms underlying this difference, providing a theoretical foundation for exploring neonatal mice as a source of inspiration for SCI repair.

### 2.2 Bulk Transcriptomic Profiling Reveals Age‐Dependent Molecular Signatures in the Early Stage of SCI

To elucidate early molecular responses to SCI across age groups, we performed transcriptomic profiling of spinal cord tissues from neonatal to adult mice at 1, 3 and 7 dpi. Principal component analysis (PCA) demonstrated clear segregation between neonatal and adult samples across time points, indicating age as a dominant factor in post‐SCI transcriptomic remodelling. Neonatal mice showed tighter PCA clustering across injury time points, indicating buffered transcriptomic perturbations during development, whereas adult mice exhibited greater molecular heterogeneity and dispersion (Figure 2A). We further assessed the dynamics of gene sets characteristic of spinal cord tissues from neonatal to adult mice. Genes associated with neurodevelopment and nerve regeneration were markedly upregulated in neonatal mice, showing transient elevation at 1–3 dpi followed by a decline (Figure 1I and J). In contrast, mitochondrial and ATP‐related gene sets were consistently higher in adults across all time points. Inflammatory gene sets were highly expressed in adult mice but only transiently activated in neonates (peaking at 1 and 3 dpi before declining), whereas adults showed a progressive upregulation. (Figure 1I and K). These results underscore fundamental age‐dependent differences in molecular response patterns after SCI.

**FIGURE 2:**
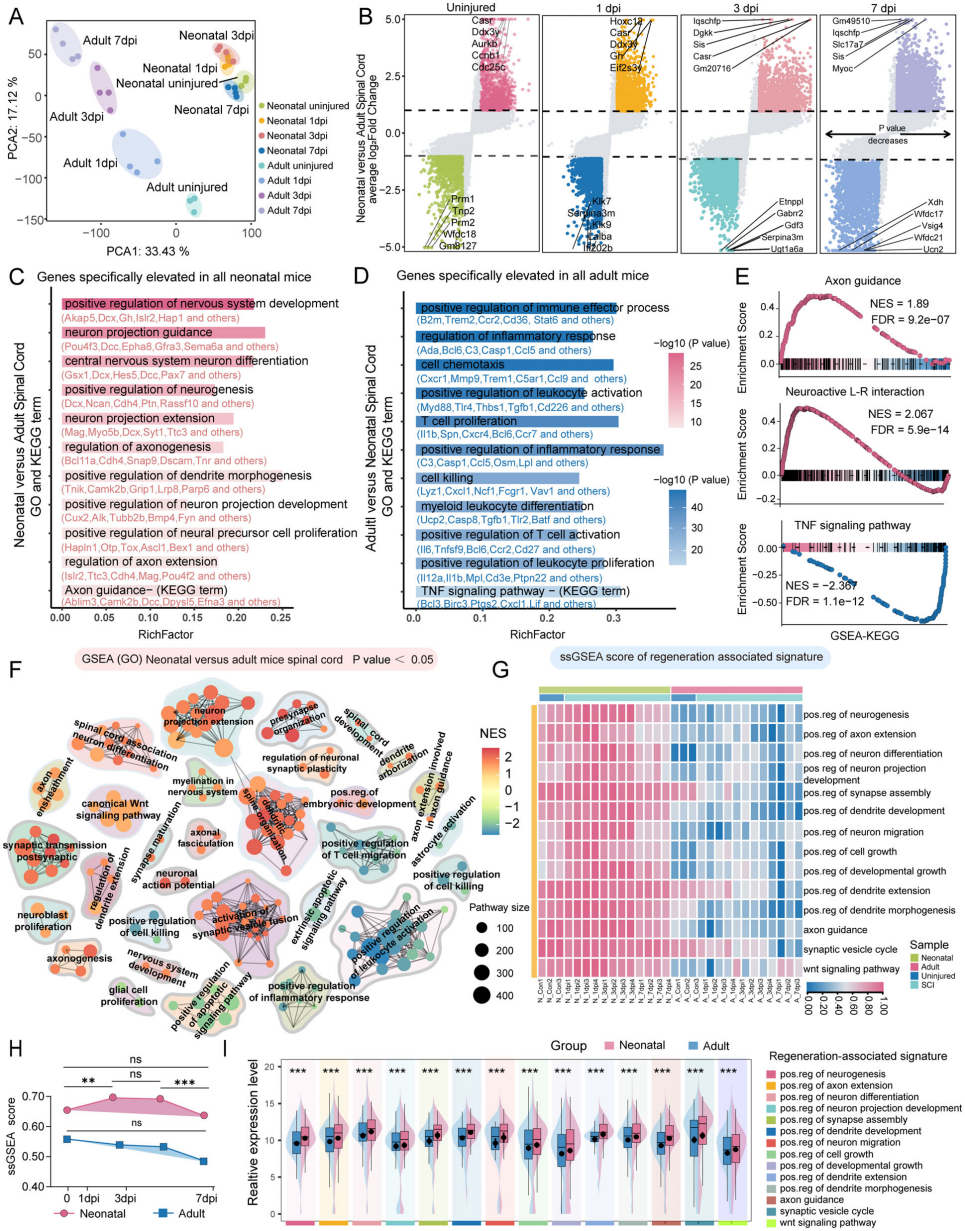
Molecular profiling of spinal cord in neonatal versus adult mice after SCI. (**A**) PCA of bulk RNA‐seq shows age‐dependent transcriptomic shifts post‐SCI. (**B**) Differential gene expression in uninjured and post‐SCI (1, 3, 7 dpi) spinal cord. (**C**–**D**) Functional enrichment of highly expressed genes in neonatal (C) and adult (D) mice. Bar color indicates –log10 (*P*‐value); length indicates enrichment factor. (**E** to **F**) GSEA of KEGG (E) and GO (F) pathways (*P* < 0.05). Dot size reflects gene number; color indicates NES. (**G**) Heatmap showing ssGSEA scores of regeneration‐related gene sets in neonatal and adult mouse spinal cords before and after injury. (**H**) Temporal dynamics of ssGSEA scores across time points. (**I**) Violin plots of regeneration‐associated genes identified via the above enrichment analysis in neonatal versus adult groups. Statistical significance was determined by one‐way ANOVA followed by Tukey's multiple comparisons post hoc test (H) or two‐tailed Student's *t* test (I). Data in H and I are presented as mean ± SD. Significance levels are indicated as ns (not significant, *P* ≥ 0.05), ^**^ *P* < 0.01, and ^***^ *P* < 0.001.

Through comparative differential expression analysis between neonatal and adult mice at each time point, we identified substantial numbers of differentially expressed genes (DEGs). Relative to adults, neonates exhibited 1230, 1628, 1834 and 3055 upregulated DEGs and 1120, 1505, 1778 and 2707 downregulated DEGs in uninjured controls and at 1, 3 and 7 dpi, respectively (log_2_FC ≥ 1 or ≤ 1, *P* < 0.05; Figure 2B). Functional enrichment analysis revealed that DEGs upregulated in all neonatal samples were enriched in regeneration‐associated processes, including nervous system development and neurogenesis, with representative genes such as *Akap5, Dcx* and *Ncan* (Figure 2C). In contrast, adult spinal cord tissues showed enrichment of inflammation‐related pathways, including immune effector processes, inflammatory response, and cell death, involving genes such as *Trem2, Ccr2* and *Cd36* (Figure 2D).

To capture broader pathway‐level changes beyond threshold‐based DEGs, we conducted gene set enrichment analysis (GSEA) using Kyoto Encyclopedia of Genes and Genomes (KEGG) and Gene Ontology (GO) databases. Axon guidance (NES = 1.89, *P* < 0.001) and neuroactive ligand–receptor interactions (NES = 2.067, *P* < 0.001) were positively enriched in neonates, while the TNF signaling pathway (NES = ‐2.367, *P* < 0.001) was negatively enriched in adults (Figure 2E). GO‐based GSEA further indicated significant enrichment of neuron projection extension and spinal cord neuron differentiation in neonates (NES = 1.82 and 1.95, respectively; *P* < 0.001), whereas adults exhibited enrichment in positive regulation of inflammatory response and cell killing (NES = ‐2.56 and ‐2.33, respectively; *P* < 0.001) (Figure 2F).

Next, we evaluated the temporal activity patterns of neurorepair pathways using ssGSEA. Pathway scores related to neuroregeneration increased in neonates after injury, peaking at 1–3 dpi before declining. Adults showed progressively decreasing scores post‐SCI, though these changes were not statistically significant (Figure 2G and H). Notably, beneficial neuroregenerative genes identified via the above enrichment analysis were generally more highly expressed in neonates than in adults (Figure 2I).

Finally, Mfuzz clustering of gene expression patterns over time revealed distinct functional transitions. In neonates, pre‐injury genes were associated with mitochondrial and respiratory functions; at 1 dpi, axon guidance and neuroregeneration were prominent; at 3 dpi, wound healing and inflammation regulation were enriched; and by 7 dpi, metabolic pathways such as glycerolipid and fatty acid metabolism were predominant (Figure S2A). In adults, pre‐injury genes were linked to neuroregeneration and axon guidance; at 1 dpi, RNA processing and translation regulation were central; and from 3 to 7 dpi, inflammatory responses and NF‐κB activation were predominant (Figure S2B).

Collectively, these findings demonstrate that, compared with adult mice which exhibit sustained inflammatory activation, neonatal mice mount a more robust transcriptional response associated with regeneration and transient inflammation resolution following SCI. This work provides mechanistic insights into age‐dependent repair processes and supports the utility of neonatal models in identifying pro‐regenerative pathways for SCI recovery.

### 2.3 Age‐Dependent Cellular Dynamics and Regenerative Heterogeneity in SCI Revealed by snRNA‐Seq

Using single‐nucleus RNA sequencing (snRNA‐seq) on 82,680 nuclei (referred to as cells hereafter for simplicity) from spinal cord lesion areas of neonatal, adult and aged C57BL/6J mice at baseline (uninjured), 3 dpi, and 7 dpi, we established a comprehensive cellular atlas following stringent quality control and dimensionality reduction (PCA/UMAP). This atlas delineated 10 distinct cell populations: oligodendrocytes, astrocytes, neurons, endothelial cells, ependymal cells, oligodendrocyte progenitor cells (OPCs), fibroblasts, immune cells, Schwann cells and pericytes (Figure 3A). Age‐stratified analysis revealed unique response patterns across developmental stages (Figure 3 B–D). Canonical marker expression (e.g., astrocytes: *Aqp4, Gfap, Slc1a3*; endothelial cells: *Vwf, Fit1, Cd34, Cdh5, Pecam1*; ependymal cells: *Dnah5/11/12*; fibroblasts: *Col1a1, Dcn, Serpinh1, Pdgfra*; neurons: *Syt1, Gap43, Dcx, Rbfox3*; oligodendrocytes: *Mog, Mag, Mbp, Plp1*; immune cells: *Ptprc, Ctss, Itgb2, P2ry12, F13a1, Ly86*; OPCs: *Tnr, Cspg4, Pdgfra, Sox10*; Schwann cells: *Prx, Mpz, Gldn, Pmp22*; pericytes: *Rgs5, Cspg4*) robustly validated cell identities and supported accurate discrimination (Figure 3E, Figure S3A and Data S3). Canonically restricted to peripheral nerves, mature Schwann cells are absent from the uninjured CNS. Detection of lesion‐resident S100β^+^/MBP^+^/Olig2^−^ cells confirms that post‐SCI Schwann cell remyelination is a true pathological event, not a tissue dissociation artifact (Figure S4).

**FIGURE 3:**
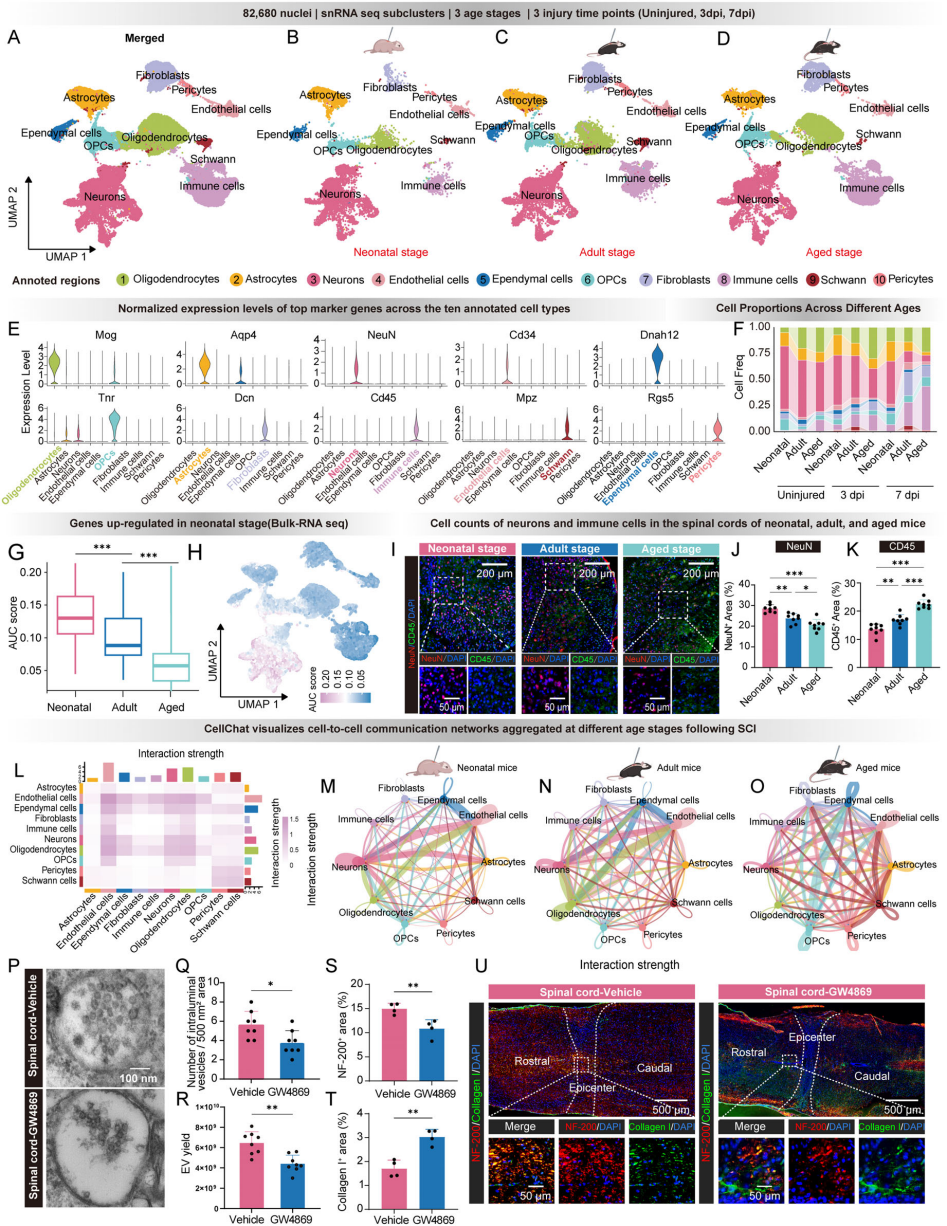
Single‐cell landscape and intercellular communication in neonatal, adult, and aged mice after SCI. (**A**) Integrated UMAP showing 10 distinct cell types. (**B–D**) Age‐specific cellular atlases for neonatal (B), adult (C), and aged (D) mice. (**E**) Violin plots of canonical marker genes per cell type. (**F**) Proportional changes of cell types across age groups and time points (uninjured, 3 dpi and 7 dpi). (**G** and **H**) AUCell scoring and UMAP visualization of neonatal‐enriched gene activity across age groups. (**I**–**K**) Immunofluorescence and quantification of NeuN^+^ neurons and CD45^+^ immune cells (*n* = 8). (**L**) Heatmap of overall communication strength between cell types post‐SCI. (**M**–**O**) Age‐specific intercellular communication networks in neonatal (M), adult (N), and aged (O) mice. Line thickness indicates interaction strength. (**P**–**R**) TEM and quantitative analysis showed GW4869 inhibited multivesicular body maturation and reduced tissue ‐ derived EVyield (*n* = 8). (**S**–**U**) GW4869 impaired tissue repair, with fewer NF‐200^+^ neurons (S) and increased Collagen I^+^ area (T), indicating compromised regeneration upon inhibition of nSC‐EV secretion (*n* = 4). Statistical significance was determined by one‐way ANOVA followed by Tukey's multiple comparisons post hoc test (G, J and K) or two‐tailed Student's *t* test (Q, R, S and T). Data are presented as mean ± SD. Significance levels are indicated as ns (not significant, *P* ≥ 0.05), ^*^ *P* < 0.05, ^**^ *P* < 0.01, and ^***^ *P* < 0.001.

Further, snRNA‐seq analysis revealed significant, age‐dependent shifts in cellular composition under both uninjured and injured conditions: neuronal and astrocyte proportions declined with age, whereas oligodendrocyte and immune cell proportions progressively increased. Post‐SCI, this trend was amplified, with neuronal proportion showing a pronounced reduction correlating with declining regenerative capacity (overall trend: neonatal, 49.2%, adult, 33.7%, aged, 19.3%), and immune cell infiltration markedly escalating, peaking at 7 dpi and intensifying with age (overall trend: neonatal: 4.2%, adult: 13.0%, aged: 29.4%) (Figure 3F), suggesting a critical role for immune recruitment in repair of SCI. Moreover, integrative analysis of bulk and snRNA‐seq identified axonal regeneration‐promoting genes exhibiting peak neonatal expression followed by age‐dependent downregulation. Area Under the Curve of cell expression (AUCell) scoring confirmed significant transcriptional attenuation of these neonatal signatures with aging at snRNA‐seq resolution (Figure 3G), indicating a progressive weakening of axonal regenerative programs at the cellular level. Spatial mapping of AUC scores onto UMAP embeddings revealed heterogeneous activity, with neurons and OPCs showing enrichment of neonatal signatures and immune cells and fibroblasts exhibiting reduced activity (Figure 3H and Figure S3B). This validated bulk findings and revealed cell type‐specific heterogeneity underlying regenerative decline. Furthermore, immunofluorescence quantification of NeuN^+^ neurons and CD45^+^ immune cells robustly confirmed the progressive age‐associated neuronal decrease and immune cell increase observed transcriptomically (Figure 3I–K).

Activity scoring of functional pathways in snRNA‐seq showed axonal regeneration‐associated pathways (e.g., neurogenesis, circuit reorganization) were most active in neonates, intermediate in adults and lowest in aged mice (Figure S5, A–F), while inflammation and cell death‐associated pathways exhibited the inverse pattern (Figure S5, G–L). Temporal analysis post‐SCI revealed distinct dynamics: neurogenesis/circuit reorganization pathways were significantly upregulated at 3 dpi versus baseline in neonates but declined by 7 dpi, whereas adults and aged mice showed significant downregulation at 3 dpi with partial recovery by 7 dpi (Figure S5, M and N). Conversely, inflammation/programmed cell death pathways peaked at 3 dpi and then declined in neonates, but showed progressive upregulation across time points in adults and aged mice (Figure S5, O and P). Cell type‐specific AUCell scoring localized the highest activity of axonal regeneration pathways to neurons and OPCs, and the lowest to immune cells (Figure S5, Q and R), while inflammation/cell death pathways were most active in immune cells and least active in neurons (Figure S5, S and T).

Together, these findings extend prior bulk analyses by providing snRNA‐seq resolution insights into age‐dependent differences in cellular composition and the dynamic regulation of functional pathways essential for neural repair after SCI, revealing attenuated neurorepair programs and amplified inflammation with aging. This work supplies critical experimental evidence delineating the age‐related molecular mechanisms underlying neural repair.

### 2.4 Neonatal‐Predominant Remodelling of Intercellular Communication Networks Drives EV‐Mediated Repair After SCI

We first characterized the landscape of intercellular communication after SCI by analyzing interaction quantity (number of signalling pathways) and intensity (functional strength of signals). All major cell types engaged in cross‐talk, with particularly robust interactions between pericytes, Schwann cells, neurons, oligodendrocytes and endothelial cells (Figure 3L). In terms of the quantity of intercellular communication, pericytes, Schwann cells, and astrocytes exhibited a relatively higher volume of intercellular communication following SCI in mice of different age groups (Figure S6A).

Striking differences emerged across age groups. Neonatal mice displayed a tightly coordinated network centred on neurons, with extensive interactions involving endothelial cells, oligodendrocytes and ependymal cells (Figure 3M and Figure S6B). In contrast, SCI in adult mice induced a more complex microenvironment, characterized by reduced communication strength between neurons and endothelial cells, alongside increased communication volume among immune cells, fibroblasts and astrocytes (Figure 3N and Figure S6C). This shift reflects heightened tissue remodelling and inflammatory regulation in mature tissue. Aged mice exhibited global communication decline, particularly in neuron‐associated pathways, aligning with their reduced regenerative capacity (Figure 3O and Figure S6D).

Building on the observed age‐specific communication patterns, pathway‐level analysis uncovered fundamental mechanistic differences underlying these network dynamics. Neonates preferentially activated neurogenic and axon guidance pathways (e.g., AGRN, SEMA6, L1CAM), supporting their superior regenerative potential (Figure S6, E and G). Conversely, adult and aged mice upregulated inflammation (e.g., SPP1, THBS) and gliosis‐related signals (e.g., IGF), indicating a trade‐off between neural repair and scar formation (Figure S6, F and G).

Next, we interrogated EVs, the dominant vehicles of cell‐cell communication, to determine their potential to encode these age‐specific networking patterns and drive differential repair outcomes. Emerging evidence suggests that tissue‐derived EVs better recapitulate the complex molecular signatures of their native microenvironment compared to culture‐conditioned EVs from isolated cell populations (Luo et al. 2023). Therefore, we systematically characterized the dynamic expression profiles of EV‐related genes in spinal cord tissues across different age groups post‐SCI (He et al. 2024). Results showed that the dynamic pattern of EV‐related gene sets aligned closely with key pathways promoting neurogenesis and inflammation regulation (Figure S6H). Specifically, in neonatal mice, the expression of EV‐related genes significantly increased at 3 dpi and declined by 7 dpi, mirroring the dynamic changes of the aforementioned neuroregeneration and inflammation‐related pathways. In contrast, in adult and aged mice, the expression of EV‐related genes progressively increased at 3 and 7 dpi, consistent with the trends of inflammation regulation and cell death‐related functions. These results suggest that EVs may play a significant role in various pathophysiological processes, especially in the early stages of SCI in mice of different ages, and their dynamic changes in neonatal mice may reflect the interaction of key mechanisms such as neuroregeneration and inflammation regulation during SCI repair.

To establish the functional necessity of nSC‐EVs in neonatal SCI repair, we pharmacologically inhibited EV secretion using GW4869 (Hu et al. 2022). Transmission electron microscopy (TEM) revealed that GW4869 treatment significantly disrupted multivesicular body maturation (Figure 3P and Q), resulting in a marked reduction in tissue ‐ derived EV yield (Figure 3R). Histological assessment demonstrated that EV secretion blockade profoundly compromised repair outcomes. Compared to vehicle controls, neonates treated with GW4869 showed significantly impaired axonal regeneration, evidenced by a reduction in NF‐200^+^ fibers, along with aggravated fibrotic scarring marked by increased Collagen I deposition at lesion sites (Figure 3S–U). These findings provide direct experimental evidence that nSC‐EVs secretion is mechanistically required for the superior regenerative capacity observed in neonatal SCI.

### 2.5 Integrated Molecular Profiling Defines Age‐Stratified Functional Landscapes of Spinal Cord Extracellular Vesicles

To elucidate age‐dependent functional dynamics of SC‐EVs, we established an integrated workflow (Figure 4A) for isolating EVs from neonatal, adult and old spinal cord tissues (nSC‐, aSC‐, oSC‐EVs) alongside primary neural stem cells (NSCs‐EVs) and neurons (Neurons‐EVs) (Figure S7, A– C). Morphological examination by TEM confirmed canonical cup‐shaped structures across all groups (Figure 4B and Figure S7D), while surface marker quantification via ExoView revealed consistent expression of CD9, CD63, and CD81 (Figure 4C and Figure S7E). Nanoparticle tracking analysis (NTA) delineated similar size distributions, with SC‐EVs exhibiting slightly larger particle sizes than cell‐derived EVs (Figure 4D and Figure S7F). Western blotting confirmed enriched EV markers (HSP90/TSG101) and showed minimal levels of the cellular contamination marker Calnexin (Figure 4E and Figure S7G). Notably, by comparing the yields of EVs from different sources under rigorously normalized conditions, it was revealed that the extraction efficiency of SC‐EVs was significantly higher than that of NSC‐EVs and Neuron‐EVs (*P* < 0.001; Figure S7H). For spinal cord tissue, cell equivalents were calculated from the wet weight of the harvested tissue. This yield advantage not only underscores the superior scalability of SC‐EVs isolation but also supports their feasibility for clinical‐grade manufacturing.

**FIGURE 4:**
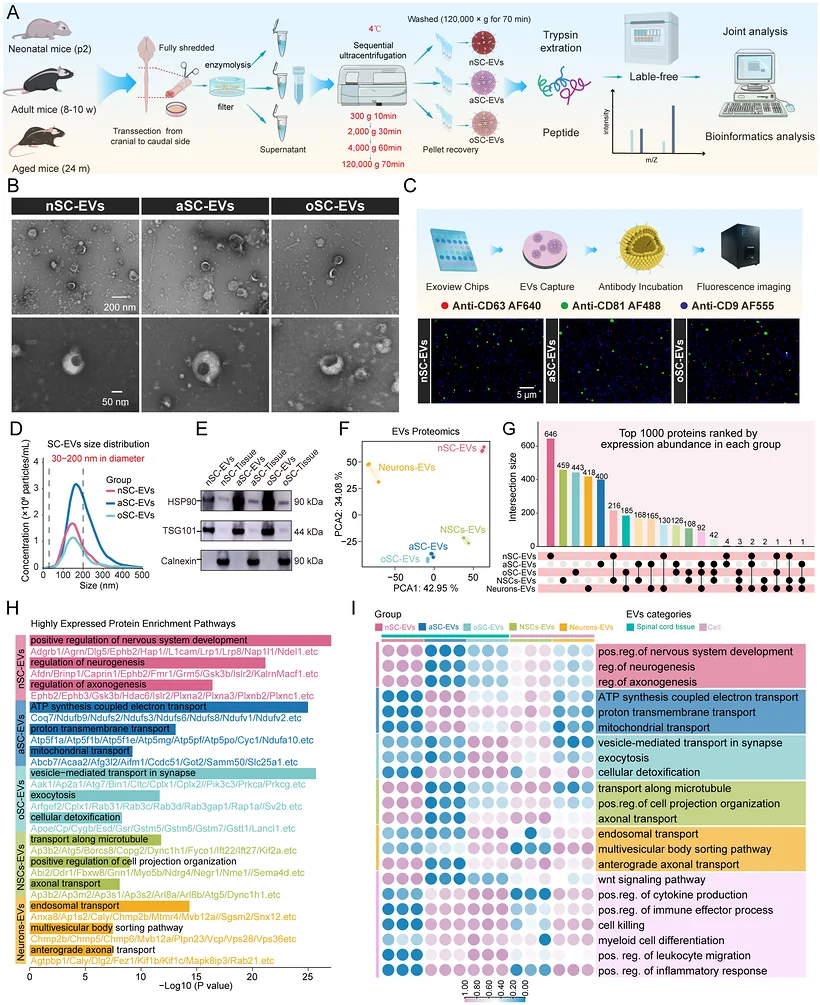
Isolation, characterization and proteomic profiling of nSC‐EVs, aSC‐EVs and oSC‐EVs. (**A**) Workflow of EV isolation, characterization and proteomic analysis. (**B**) TEM images showing typical cup‐shaped morphology of SC‐EVs. (**C**) ExoView detection of EV markers (CD9, CD63, CD81). (**D**) NTA indicating size distribution. (**E**) Western blotting confirmed the enrichment of EV markers (HSP90, TSG101), with barely detectable Calnexin. (**F**) PCA of proteomic profiles from multiple EV types. (**G**) Overlap of the top 1000 highly abundant proteins across tissue‐derived and cell‐derived EV preparations. (**H**) Functional annotation of source‐specific enriched proteins. (**I**) Comparative analysis of functional activities of enriched proteins.

To characterize cargo differences among nSC‐EVs, aSC‐EVs and oSC‐EVs, we performed miRNA profiling. A total of 1,755 miRNAs were detected, comprising 458 novel miRNAs and 1297 known mmu‐miRNAs. PCA revealed prominent age‐dependent cargo remodelling, with nSC‐EVs distinctly separated from aSC‐EVs and oSC‐EVs (Figure S8A). Differential expression analysis (|log_2_FC| ≥ 1, *P* < 0.05) identified 270 upregulated and 192 downregulated miRNAs in nSC‐EVs relative to aSC‐EVs; the aged‐adult comparison exhibited minimal variation with only 20 altered miRNAs. By contrast, the oSC‐EV versus nSC‐EV comparison uncovered 182 upregulated and 260 downregulated miRNAs (Figure S8B). To further refine core miRNA signatures, stringent filtering (|log_2_FC| ≥ 2, *P* < 0.05) defined 85 Neo‐common‐down and 35 Neo‐common‐up miRNAs consistently altered in nSC‐EVs versus both aSC‐EVs and oSC‐EVs (Figure S8, C and D). To investigate the functional impact of these miRNA alterations, we predicted downstream target mRNAs and performed enrichment analysis. Cross‐analysis of five prediction databases retrieved 140 derepressed mRNAs enriched in cell growth, angiogenesis, neural precursor proliferation and tissue regeneration (Figure S8E and Figure S9A), alongside 21 suppressed mRNAs functionally linked to immune/inflammation responses and glial differentiation (Figure S8F and Figure S9B). Collectively, these data demonstrate that nSC‐EVs possess a unique pro‐regenerative miRNA landscape sharply divergent from both aSC‐EVs and oSC‐EVs, whereas the molecular disparity between adult and aged EVs remains limited.

In parallel, proteomics was performed to characterize the protein cargo of nSC‐EVs, aSC‐EVs, and oSC‐EVs. Proteomics uncovered fundamental compositional divergence among EV sources. PCA segregated tissue‐derived EVs from cell‐derived counterparts, with nSC‐EVs forming a discrete cluster distinct from both aSC‐EVs and oSC‐EVs (Figure 4F). Analysis of the top 1000 proteins revealed source‐specific signatures: nSC‐EVs uniquely enriched 646 proteins compared to 400 in aSC‐EVs and 443 in oSC‐EVs (Figure 4G). Age‐dependent functional specialization emerged, where nSC‐EVs prioritized neuroregulatory functions through axonogenesis effectors (e.g., Gsk3b, L1cam), while aSC‐EVs shifted toward mitochondrial energy metabolism (Atp5f1a, Ndufb9) and oSC‐EVs upregulated synaptic vesicle cycling (e.g., Acaa2, Aak1) alongside detoxification machinery (e.g., Apoe, Cygb). Complementary specialization occurred in cell‐derived EVs: NSCs‐EVs conserved microtubule transport components (e.g., Ap3b2, Atg5), whereas Neurons‐EVs enriched multivesicular body sorting factors (e.g., Chmp2b, Mvb12a) (Figure 4H). Activity scoring revealed that nSC‐EVs harbored the greatest neurogenic potential, with Wnt and neurogenesis‐related pathway activities significantly elevated compared to oSC‐EVs, in stark contrast to the pro‐inflammatory signature dominant in aged groups (Figure 4I). These findings demonstrate that nSC‐EVs possess markedly superior neurogenesis‐promoting capacity than other EV sources.

Subsequent differential proteomics (|log_2_FC| ≥ 1, *P* < 0.05) corroborated this developmental reprogramming: nSC‐EVs versus aSC‐EVs comparisons revealed 177 upregulated neurogenic proteins governing neural precursor proliferation, axon guidance, and dendritic morphogenesis (Figure S10A–B). Conversely, oSC‐EVs versus aSC‐EVs exhibited 30 elevated inflammatory mediators regulating lymphocyte activation and oxidative stress responses (Figure S10A and C). GSEA further validated these divergent trajectories: nSC‐EVs activated nervous system development pathways, whereas oSC‐EVs upregulated chemokine signalling (Figure S10D–E). To resolve proteomic dynamics beyond top‐abundance proteins, we performed Mfuzz clustering of 7142 proteins across SC‐EVs, identifying three ontogenetic trajectories (Figure S11, Data S4). nSC‐EVs were defined by a 3463‐protein cluster enriched in neurodevelopmental regulators (e.g., Bdnf, Gap43, Dcx), functionally orchestrating synaptic organization, nervous system development and axonogenesis. aSC‐EVs featured a distinct 1318‐protein module dominated by energy metabolism effectors (e.g., Atp5if1, Atp5mc2) driving cellular respiration and ATP biosynthesis. In contrast, oSC‐EVs carried a 2361‐protein signature specializing in synaptic vesicle cycling (e.g., Cacna1a, Syt1) and neurotransmitter release machinery, with substantial expression overlap in aSC‐EVs, indicating progressive functional drift during aging (Figure S11).

To integrate miRNA and proteomic datasets, we intersected predicted target genes of miRNAs differentially expressed in nSC‐EVs with age‐stratified proteomic signatures: 3463 neonatal‐enriched versus 3679 adult/aged‐enriched proteins. Targets were compiled from experimentally validated interactions (miRTarBase, TarBase) and consensus predictions across TargetScan, miRDB, miRanda, and DIANA‐microT, yielding a multi‐layered miRNA‐mRNA‐protein regulatory network of genes de‐repressed at the miRNA level and highly expressed at the protein level. As shown in Figure S12A–B, de‐repressed targets in nSC‐EVs yielded 111 unique genes (miRTarBase: 31; TarBase: 109 after union and deduplication), markedly exceeding adult/aged groups (27 unique genes; miRTarBase: 9; TarBase: 24). Functional enrichment revealed that nSC‐EV intersection molecules were significantly enriched in neurogenic pathways—neurogenesis, axonogenesis and cell junction assembly—whereas a/oSC‐EVs showed only weak enrichment in macrophage cytokine production and GTPase activity (Figure S12C). These findings indicate that neonatal EVs concurrently relieve suppression of repair‐related proteins and actively suppress immune‐inflammatory and glial differentiation pathways through specific miRNA upregulation, maintaining a pro‐regenerative microenvironment.

Furthermore, building upon nSC‐EV proteomic signatures, integrated transcriptomic analyses revealed spatially and temporally restricted expression patterns. Bulk RNA‐seq demonstrated significantly elevated expression of nSC‐EVs signature genes in uninjured neonatal versus the adult/aged spinal cords (Figure S13A), a pattern recapitulated at snRNA‐seq resolution where AUCell scores decreased progressively with age (Figure S13B) and peaked in neurons (Figure S13C). Post‐injury dynamics in neonates revealed transient upregulation of these genes at 3 dpi followed by decline at 7 dpi (Figure S13D), precisely aligning with regenerative windows (Figure 2H). Cellular deconvolution confirmed neuron/OPCs enrichment for nSC‐EVs signatures versus fibroblast/astrocyte/immune cell localization of aSC‐EVs/oSC‐EVs (Figure S13E–G). Intersectional analysis identified 39 neonatal‐elevated molecules (Data S5), of which 20 representative molecules (Gap43, Tubb5, Ndn, etc.) were highlighted, showing coordinate overexpression in neonatal tissue transcriptomes and EVs proteomes (Figure S13H), and were functionally enriched in axon regeneration and synaptogenesis pathways (Figure S13I). The research demonstrated that the highly expressed proteins in nSC‐EVs are closely consistent with the molecular profile of the neonatal spinal cord.

### 2.6 Wnt/β‐Catenin Signalling Mediates nSC‐EV ‐Induced NSC Proliferation

Building upon the established axonal regenerative potential of nSC‐EVs, we first confirmed their cellular uptake. Fluorescent tracing using PKH26‐labeled EVs demonstrated efficient neuronal internalization, with significant cytoplasmic accumulation and colocalization with β‐III‐Tubulin positive somata after 24‐h coculture (Figure S14A). This foundational evidence established the cellular interaction basis for subsequent functional investigations.

Transcriptomic profiling of NSCs treated with nSC‐EVs versus aSC‐EVs revealed profound molecular specialization. Bulk RNA sequencing identified nSC‐EVs specific upregulation of some functional modules: cell cycle regulators governing DNA replication, axon guidance, synapse assembly and Wnt signalling components (Figure 5A and B). Heatmap analysis further revealed that, compared to the aSC‐EVs group, the nSC‐EVs treatment group exhibited consistent upregulation of representative genes for neural progenitor proliferation markers (*Nes, Sox10, Hapln2*) (Figure 5C), Wnt pathway activators (*Wnt5a, Hhex, Lgr5*) (Figure 5D), and positive regulators of neurogenesis (*Bmp7, Pax6, Neurod1*) (Figure S14B). These results suggest that nSC‐EVs may influence the repair process of SCI by affecting neural progenitor proliferation and neurogenesis.

**FIGURE 5:**
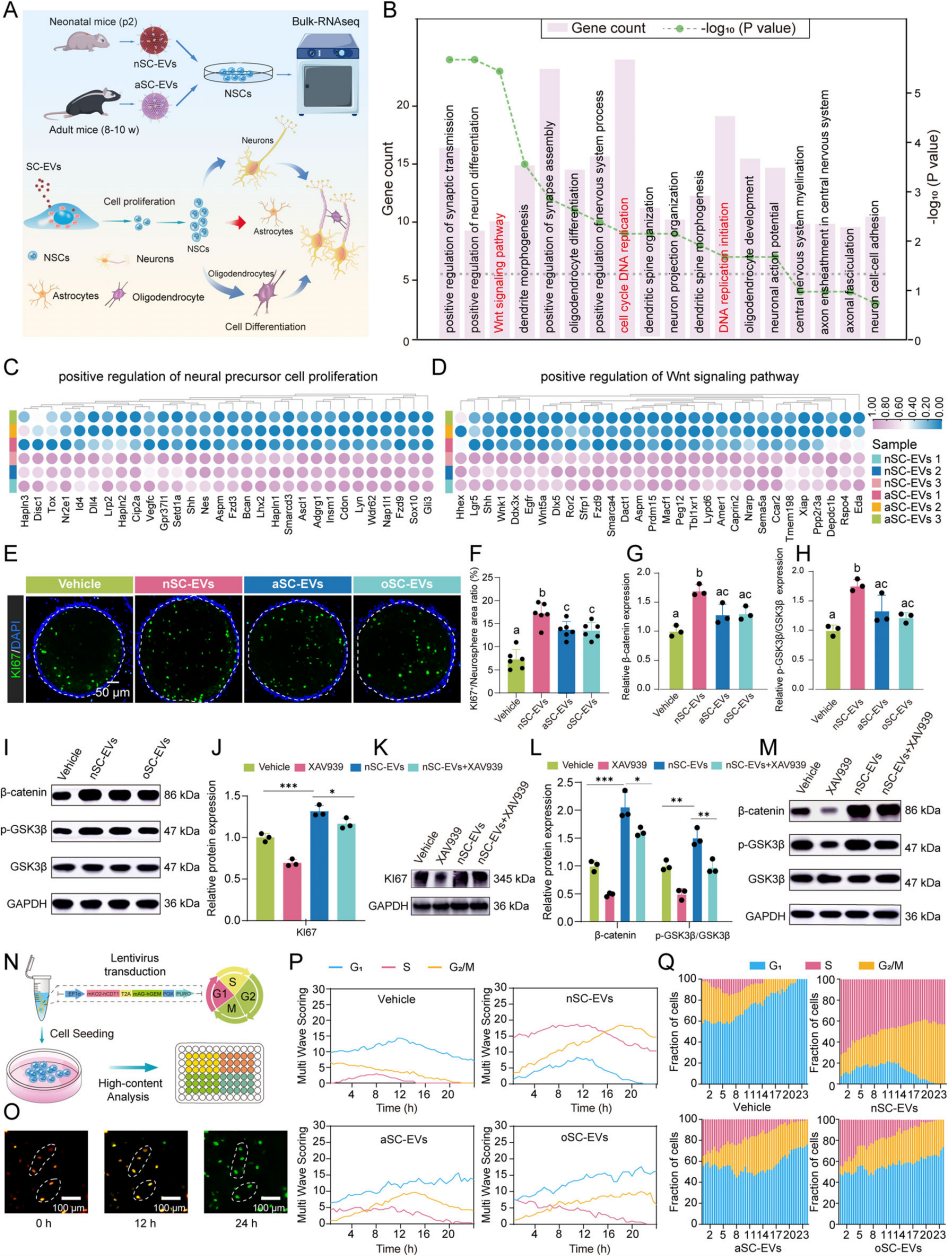
nSC‐EVs promote NSC proliferation via the Wnt/β‐catenin pathway. (**A**) Schematic of experimental design. (**B**) Enrichment analysis of genes upregulated by nSC‐EVs versus aSC‐EVs (|log2FC| ≥ 1, *P* < 0.05). Dot height and bar length indicate gene count and pathway significance, respectively. (**C** and **D**) Expression changes of genes related to neural precursor cell proliferation (C) and Wnt signalling activation (D). (**E–F**) Proliferation marker KI67 detected by immunofluorescence (*n* = 6). (**G**–**I**) Key molecular changes in the Wnt/β‐catenin pathway (*n* = 3). (**J**–**K**) KI67 expression was decreased by XAV939 and restored by nSC‐EVs, which was abolished upon co‐treatment with XAV939 (*n* = 3). (**L** and **M**) Rescue experiments confirming the essential role of Wnt/β‐catenin signalling (*n* = 3). (**N**) Schematic of the FUCCI system for real‐time cell cycle monitoring. (**O**) Representative time‐lapse images of FUCCI‐labeled NSCs showing distinct fluorescent colors indicating different cell cycle phases (red: G_1_; yellow: S; green: G_2_/M). (**P**) FUCCI ‐ basedscoring of cell cycle phase transitions across treatments (*n* = 3). (**Q**) Proportion of NSCs in each cell cycle phase after different treatments (*n* = 3). Statistical significance was determined by Kruskal–Wallis test with FWE correction for multiple comparisons followed by post hoc pairwise between‐group tests (F, G, H, J and L). Data are presented as median (interquartile range). For panels F, G and H, groups with no shared letters indicate significant differences between them. For panels J and L, significance levels are indicated as ^*^ *P* < 0.05, ^**^ *P* < 0.01, and ^***^ *P* < 0.001.

Functional validation confirmed these transcriptomic predictions. EdU incorporation assays revealed that nSC‐EVs increased NSC proliferation relative to vehicle controls (Figure S15A and B). Concomitant upregulation of the proliferation marker KI67 was confirmed by immunofluorescence, with the cell positive area ratio increased relative to that of the vehicle group (Figure 5E‐F). Similarly, the western blotting results confirmed that nSC‐EVs activate the Wnt/β‐catenin pathway by upregulating β‐catenin and enhancing GSK‐3β phosphorylation (Figure 5G–I). To further confirm the crucial role of the Wnt pathway in this process, rescue experiments were carried out. When NSCs were treated with a Wnt pathway inhibitor, the promotional effect of nSC‐EVs on NSC proliferation was significantly attenuated (Figure 5J–M). This directly confirms the central role of Wnt pathway activation in nSC‐EV‐ mediated NSC proliferation and further reveals the underlying molecular mechanism.

To resolve temporal dynamics, we employed FUCCI cell‐cycle reporters (Figure 5N). Using the real‐time dynamic monitoring capability of the FUCCI system along with time‐lapse fluorescence imaging (Figure 5O), the dynamic transition of the cell cycle from the G_1_ phase to the S/G_2_/M phase in NSCs under diverse treatment conditions was clearly shown. FUCCI ‐ based scoring analysis (Figure 5P) and cell proportion analysis in different cell cycle phases (Figure 5Q) demonstrated that nSC‐EVs considerably accelerated the transition of NSCs from the G_1_ phase to the S and G_2_/M phases compared to other groups. Specifically, the nSC‐EVs group showed a significantly higher conversion efficiency from G_1_ to S phase. This suggests that nSC‐EVs might speed up the proliferation of NSCs by promoting the activation of key cell cycle regulation checkpoints. This cell‐cycle acceleration provides a kinetic basis for the observed proliferation enhancement.

Collectively, these data indicate that nSC‐EVs activate the Wnt/β‐catenin signalling pathway, accelerate cell cycle progression and promote the expansion of NSCs. The consistency and robustness of transcriptional predictions, functional validation and cell cycle dynamics analysis highlight the reliability of nSC‐EVs as mediators of NSC proliferation.

### 2.7 Neurogenic Effects of nSC‐EVs Are Mediated *via* the Agrin Protein

We integrated 7127 genes and 463 proteins from the transcriptome of neonatal mouse spinal cord tissue and SC‐EVs proteomics, identifying 59 molecules shared with the neuroregeneration‐related gene set (GO:0050769). We randomly selected 30 representative ones and displayed their expression trends in a heatmap (Figure 6A and B). The results showed these molecules were highly expressed in neonatal mouse samples. We treated DRG explants and individual neurons with nSC‐EVs, aSC‐EVs and oSC‐EVs for 48 h. Compared to the other groups, the DRG neurons in the nSC‐EVs group displayed significantly enhanced axonal growth rates (Figure 6C and D). Specifically, the DRG explants exhibited a greater growth rate than that of  the control group (Figure 6E). Moreover, individual neurons in the nSC‐EVs group exhibited a significantly higher number of connection nodes than those in the other groups (Figure 6F). These results indicate that nSC‐EVs exhibit superior efficacy in promoting axonal growth compared to aSC‐EVs and oSC‐EVs.

**FIGURE 6:**
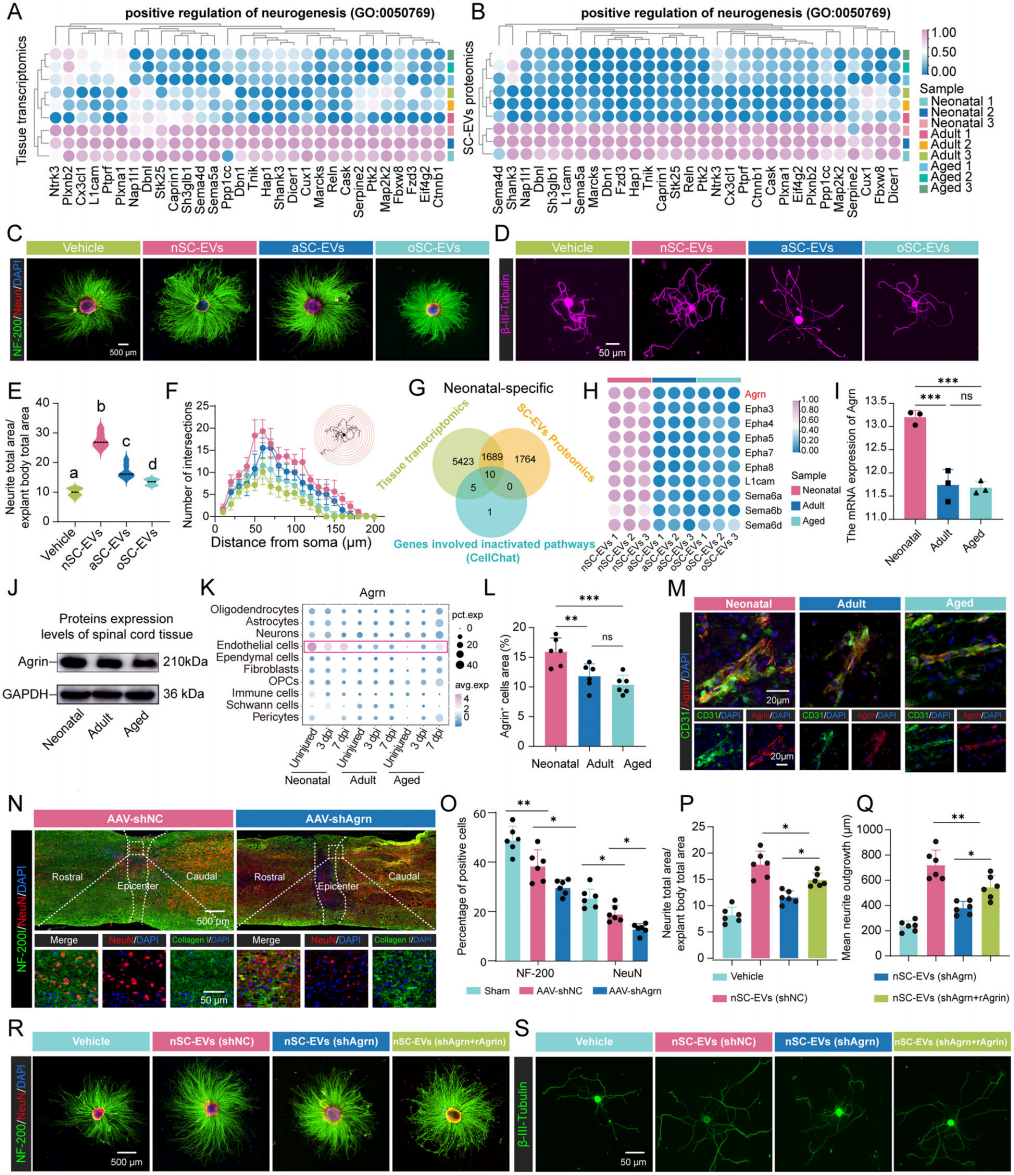
nSC‐EVs promote neuro‐regeneration and axonal growth, with Agrin (Agrn) identified as a key molecule. (**A** and **B**) Intersection analysis of spinal cord transcriptomics (A) and SC‐EVs proteomics (B), with heatmaps showing expression trends of neurogenesis‐related molecules across age groups. (**C**–**F**) SC‐EVs promote axonal growth in DRG explants (C and E) and individual neurons (D and F) (*n* = 6). (**G**) Venn diagram of significantly upregulated pathways from transcriptomic, proteomic, and cell communication data in neonatal mice. (**H**) Expression levels of overlapping molecules in SC‐EVs across age groups. (**I** and **J**) Agrin (*Agrn*) expression in spinal cord tissue at mRNA (I) (*n* = 3) and protein (J) levels. (**K**) Temporal and cell type‐specific expression of *Agrn* after SCI in mice of different ages. (**L** and **M**) Immunofluorescence and quantification of Agrin in endothelial cells across age groups (*n* = 6). (**N** and **O**) Effect of Agrin (*Agrn*) knockdown on SCI repair in neonatal mice (*n* = 6). (**P**–**S**) Impaired ability of Agrin (*Agrn*) knockdown nSC‐EVs to promote neurite outgrowth in DRG explants (P and R) and individual neurons (Q and S), rescued by reconstitution with rAgrin (*n* = 6). Groups sharing no common letters differ significantly (*P* < 0.05). Statistical significance was determined by Kruskal–Wallis test with FWE correction for multiple comparisons followed by post hoc pairwise between‐group tests (I) or one‐way ANOVA followed by Tukey's multiple comparisons post hoc test (E, L, O, P and Q). Data in panel I are presented as median (interquartile range); data in panels E, L, O, P and Q are presented as mean ± SD. For panel E, groups with no shared letters indicate significant differences between them. For panels I, L, O, P and Q, significance levels are indicated as ns (not significant, *P* ≥ 0.05), ^*^ *P* < 0.05, ^**^ *P* < 0.01, and ^***^ *P* < 0.001.

Systematic integration of tissue transcriptomics, EVs proteomics, and cell communication data pinpointed Agrin (*Agrn*) as the core effector molecule. Among the 10 key neurogenesis molecules involved in the AGRN, L1CAM, EPHA and SEMA6 signalling, Agrin (*Agrn*) displayed a prominent neonatal stage‐specific expression profile. Specifically, the protein level of Agrin in nSC‐EVs was 1.98‐fold and 2.03‐fold higher than in aSC‐EVs and oSC‐EVs, respectively (Figure 6H and Figure S16A). Consistent age‐dependent decline occurred at transcriptional (Figure 6I) and protein levels in spinal cord tissues (Figure 6J). Analysis following post‐SCI revealed that the expression level of *Agrn* in the neonatal group was significantly higher than that in the adult group at 1, 3 and 7 dpi (Figure S16B). Furthermore, snRNA‐seq resolution uncovered endothelial‐specific *Agrn* enrichment in neonatal spinal cords (Figure 6K). *Agrn* ^+^ endothelial cells demonstrated elevated positive regulation of neurogenesis (Figure S16C) alongside enhanced EV‐related gene signatures (Figure S16D). Immunofluorescence results showed that the expression of Agrin in spinal endothelial cells of neonatal mice was 1.35 and 1.53 times higher than that of adult and aged mice, respectively (Figure 6L and M). To directly assess the functional significance of endothelial cell–derived Agrin, we collected conditioned medium from primary neonatal and adult spinal cord endothelial cells (Figure S16E–G) and applied it to DRG neuron cultures in a four‐group experimental design: (i) control: conditioned medium from adult endothelial cells; (ii) positive control: conditioned medium from neonatal endothelial cells; (iii) blockade: conditioned medium from neonatal endothelial cells plus Agrin‐neutralizing antibody; and (iv) rescue: conditioned medium from adult endothelial cells plus recombinant Agrin (rAgrin). Conditioned medium from neonatal endothelial cells significantly enhanced neurite outgrowth compared with that from adult endothelial cells. Critically, this pro‐growth effect was partially attenuated upon Agrin blockade, demonstrating necessity. Conversely, supplementation of adult endothelial conditioned medium with rAgrin partially restored its neurite‐promoting capacity, confirming sufficiency (Figure S16H–K). Collectively, these results establish that Agrin is a key functional mediator of the neurotrophic activity secreted by neonatal endothelial cells.

Finally, to investigated whether nSC‐EVs promote nerve growth *via* Agrin, we knocked down the *Agrn* gene in neonatal mice to assess SCI repair phenotypes, and isolated EVs from spinal cord. AAV9/BBB‐mediated Agrin (*Agrn*) knockdown, with maternal delivery at E11.5, led to reductions in both spinal cord Agrin (*Agrn*) mRNA and protein levels at postnatal day 7 (Figure S17A–C). In the SCI model, compared to the control group, mice with Agrin (*Agrn*) knockdown exhibited significantly impaired spinal cord repair function, as evidenced by a reduction of approximately 22.6% in NF‐200 fluorescence signal and 31.9% in NeuN fluorescence signal (Figure 6N and O). Western blotting confirmed that Agrin (*Agrn*) knockdown decreased Agrin protein in nSC‐EVs (Figure S17D). To construct an overexpression rescue system, we reconstituted Agrin‐deficient nSC‐EVs with recombinant Agrin (rAgrin) using a sonication‐based loading protocol as previously described. Comprehensive physicochemical characterization confirmed successful protein incorporation with preserved vesicle integrity, size homogeneity and canonical exosomal marker expression (Figure S17E–H and Table 3).

Functionally, rAgrin‐loaded nSC‐EVs partially rescued the impaired neurogenic activity of Agrin‐deficient EVs. In both DRG explants and dissociated neurons, nSC‐EVs (sh*Agrn*) markedly reduced neurite outgrowth compared with controls, whereas reconstitution with rAgrin restored outgrowth, though not fully to control levels (Figure 6 P–S). These results firmly establish Agrin as a key functional mediator of the neurogenic activity of nSC‐EVs.

### 2.8 ACPP‐Functionalized nSC‐EVs Enhance Targeted Delivery and Nerve Regeneration in Adult Mice With SCI

To achieve lesion‐specific delivery of nSC‐EVs in SCI, we engineered their surface with ACPPs. These constructs feature a poly‐anionic inhibitory domain, poly‐cationic cell‐penetrating peptide (CPP) and matrix metalloproteinase (MMP)‐cleavable linker (Figure S18A). Mass spectrometry confirmed ACPP conjugation (EEEEEEEEEEPLGLAGR), with data‐independent acquisition (DIA) fragment ion spectra validating peptide integrity (Figure S18B). Molecular docking demonstrated specific hydrogen‐bond interactions between ACPP and MMP2/MMP9 (Figure S18C), supporting MMP‐responsive CPP exposure at the SCI lesion.

*In vivo* tracking revealed enhanced lesion targeting of ACPP@nSC‐EVs, exhibiting higher fluorescence intensity at SCI sites versus unmodified nSC‐EVs 12 h post‐injection (Figure 7B–D). Ex vivo analysis confirmed preferential accumulation of ACPP@nSC‐EVs in spinal cord lesions with concomitant elevation in brain uptake, contrasting with the hepatic‐dominated distribution of unmodified EVs (Figure S18D–I).

**FIGURE 7:**
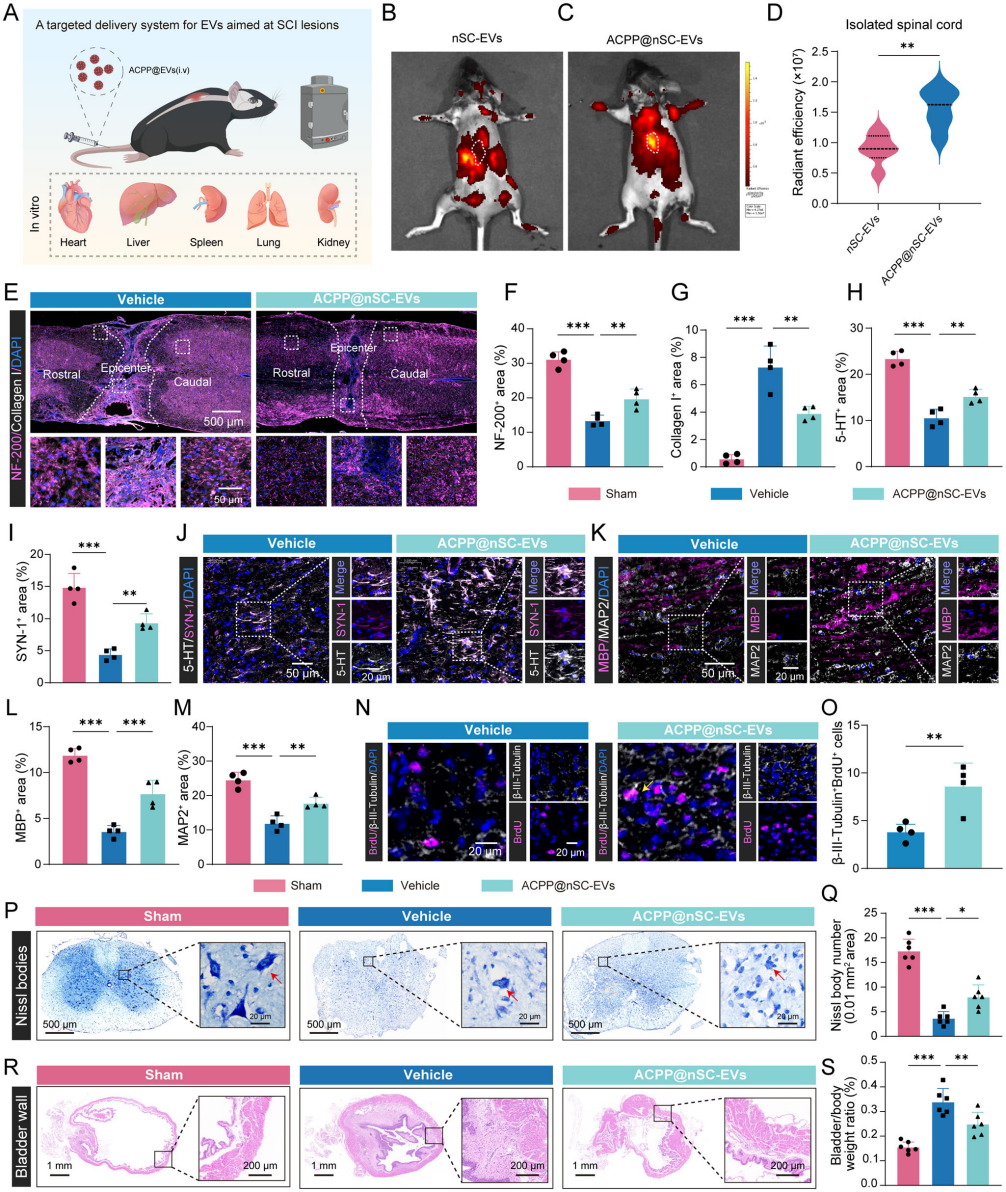
ACPP@nSC‐EVs enhanced tissue repair after SCI in adult mice. (**A**–**D**) In vivo fluorescence imaging showed targeted accumulation of DiR‐ACPP@nSC‐EVs at the injury site 12 h post‐injection, compared to DiR‐nSC‐EVs (*n* = 6). (**E**–**G**) At 42 dpi, ACPP@nSC‐EVs promoted axonal regeneration of NF‐200^+^ fibers (F) and reduced collagen I^+^ fibrotic scarring (G) (*n* = 4). (**H**–**J**) Increased immunofluorescence of 5‐HT^+^ (H) and SYN‐1^+^ (I) signals, indicating enhanced serotonergic innervation and synaptic remodelling (*n* = 4). (**K**–**M**) Improved myelination (MBP, L) and neuronal structural integrity (MAP2, M) following treatment (*n* = 4). (**N** and **O**) Increased abundance of β‐III‐Tubulin^+^/BrdU^+^ double ‐ positive newborn neurons following ACPP@nSC‐EVs treatment (*n* = 4). (**P** and **Q**) Nissl staining revealed improved tissue integrity post‐treatment (*n* = 6). (**R** and **S**) H&E staining of bladder tissue showed structural recovery (*n* = 6). Statistical significance was determined by two‐tailed Student's *t* test (D, O) or one‐way ANOVA followed by Tukey's multiple comparisons post hoc test (F, G, H, I, L, M, Q, S). Data in all above panels are presented as mean ± SD. Significance levels are indicated as ns (not significant, *P* ≥ 0.05), ^*^ *P* < 0.05, ^**^ *P* < 0.01, and ^***^ *P* < 0.001.

Comprehensive biosafety assessment showed no acute toxicity. Histopathology of major organs revealed normal architecture without inflammation (Figure S19A), while liver/kidney function markers (ALT, AST, TP, CREA) remained within physiological ranges (Figure S19B–E). Hemolysis assays confirmed excellent biocompatibility (OD value comparable to PBS controls; Figure S19F).

Therapeutic evaluation at 42 dpi demonstrated multi‐faceted nerve regeneration. ACPP@nSC‐EVs treatment significantly enhanced NF‐200^+^ axonal continuity while concurrently suppressing fibrotic scarring (Collagen I^+^ area) (Figure 7E–G). Synaptic reconstruction was markedly augmented, evidenced by 1.44‐fold and 2.12‐fold higher numbers of 5‐HT^+^ and SYN‐1^+^ cells relative to Vehicle controls, respectively (Figure 7H–J). Furthermore, remyelination advanced substantially, with the number of MBP^+^  cells increased to 2.15‐fold of the control level, paralleled by MAP2^+^ neuronal structural integrity increased to 1.49‐fold relative to vehicle controls (Figure 7K–M). To investigate whether ACPP@nSC‐EVs enhance neurogenesis by promoting the proliferation of NSCs, we performed BrdU and β‐III‐Tubulin immunostaining experiments. The results showed that the number of BrdU^+^/β‐III‐Tubulin^+^ cells in the treatment group was significantly higher than that in the vehicle group (Figure 7N and O).

These regenerative outcomes were corroborated by Nissl staining showing increased neuronal density with abundant Nissl bodies (Figure 7P and Q), and functional recovery manifested through normalized bladder wall morphology with restored detrusor muscle organization (Figure 7R and S). Collectively, ACPP@nSC‐EVs demonstrate potent therapeutic efficacy in SCI through targeted delivery and multi‐dimensional neural repair.

### 2.9 Therapeutic Effects of ACPP@nSC‐EVs on Functional Recovery After SCI

Behavioural and electrophysiological analyses demonstrated that ACPP@nSC‐EVs treatment significantly improved functional recovery in SCI mice. By 42 dpi, the ACPP@nSC‐EVs treated group exhibited enhanced hindlimb locomotor function, with mean BMS scores increasing from 3.2 to 5.3 compared to vehicle group (Figure 8A). Electromyographic recordings further confirmed functional restoration, showing increased motor evoked potential (MEP) amplitude and reduced latency in the treated group (Figure 8B‐E), indicative of improved nerve conduction.

**FIGURE 8:**
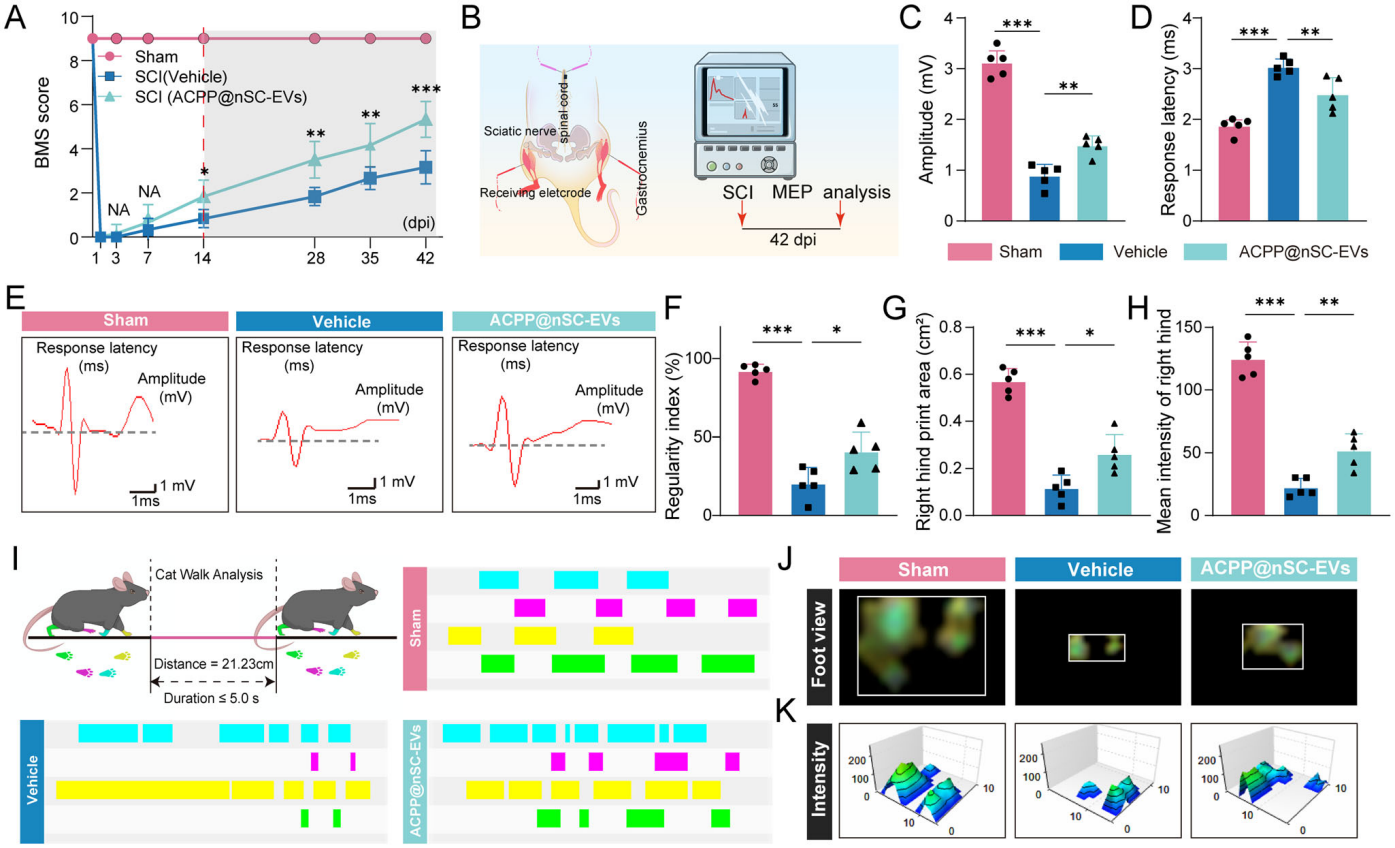
Electrophysiological and behavioural recovery after ACPP@nSC‐EVs treatment in SCI mice. (**A**) Improved hindlimb motor function over time, assessed by BMS scoring at uninjured baseline and 1, 3, 7, 14, 28, 35, 42 dpi. (B) Schematic of electromyography setup. (**C**–**E**) Motor‐evoked potential (MEP) recordings showing increased amplitude (C) and reduced latency (D) at 42 dpi (*n* = 5). (**F**–**K**) Gait analysis revealed enhanced regularity index (F), contact area (G) and weight‐bearing capacity (H) in treated mice (*n* = 5). Representative gait patterns (I), footprints (J), and intensity maps (K) are shown. A linear mixed‐effects model was used for repeated BMS measurements, followed by Sidak‐corrected post‐hoc pairwise comparisons (A). Statistical significance was determined by one‐way ANOVA followed by Tukey's multiple comparisons post hoc test (C, D, F, G and H). Data are presented as mean ± SD. Significance levels are indicated as ns (not significant, *P* ≥ 0.05), ^*^ *P* < 0.05, ^**^ *P* < 0.01, and ^***^ *P* < 0.001.

Gait analysis using the CatWalk XT system revealed that the ACPP@nSC‐EVs intervention enhanced motor coordination, as evidenced by a higher regularity index compared to controls (Figure 8F and I). Experimental data revealed an increase in the contact area and average intensity of pressure distribution in the right hind paw prints of animals (Figure 8G and H). These changes indicate a significant enhancement in the weight‐bearing capacity of the right hind paw, highlighting the potential of ACPP@nSC‐EVs in improving motor function in mice. Additionally, stride cycle regularity and swing‐stance phase ratios were optimized (Figure 8I), while plantar pressure distribution exhibited improved symmetry and contact area (Figure 8J and K).

These findings collectively indicate that ACPP@nSC‐EVs promote functional recovery after SCI by restoring nerve conduction and motor coordination.

## 3 Discussion

SCI represents a debilitating disorder of the CNS, characterized by often irreversible motor and sensory impairments. Current therapeutic strategies remain limited in effectively promoting spinal cord regeneration and functional recovery, highlighting an urgent need to explore novel regenerative interventions. In this study, we demonstrate that the efficiency of regenerative program initiation and the kinetics of inflammation resolution are critically regulated in an age‐dependent manner, governing the spatiotemporal dynamics of SCI repair. We further identify nSC‐EVs as key molecular carriers that convey youth‐associated regenerative signals, and we leverage a bioengineered delivery platform—ACPP@nSC‐EVs—to enhance targeted therapeutic intervention. Our findings establish a novel mechanistic framework underlying age‐related disparities in repair competence and offer translatable insights for the treatment of SCI.

It is well established that lower vertebrates—such as axolotls, zebrafish and African clawed frogs—retain robust regenerative capacities following SCI, even in adulthood, achieving scarless healing and functional recovery (Lee‐Liu et al. 2017; Sabin et al. 2019; Wehner et al. 2017). In contrast, mammals have largely lost the ability to regenerate complex tissues. Notably, neonatal mice and rats preserve a transient capacity for spinal cord repair, which sharply declines with maturation (Li et al. 2020; Miya et al. 1997). We observed a progressive age‐dependent reduction in repair efficiency, accompanied by elevated release of pro‐inflammatory factors and increased markers of cell death. Temporal profiling revealed that neonatal mice exhibit a narrow, high‐efficacy regenerative window during early injury phases, whereas adult and aged animals display sustained activation of inflammatory and cell death pathways. This suggests an age‐dependent imbalance between neuroprotective mechanisms and detrimental inflammatory responses may underlie the decline in regenerative capacity—a notion corroborated by recent work from Grégoire Courtine's group, whose Tabulae Paralytica revealed increased immune cell infiltration and impaired neural repair in older mice (Skinnider et al. 2024).

At the cellular level, snRNA‐seq atlases of post‐SCI tissues across age groups revealed pronounced declines in neuronal proportions with advancing age, particularly at 3 dpi and 7 dpi, consistent with reported age‐related neurodegenerative changes and diminished regenerative potential. These findings align with neuroimaging studies documenting age‐dependent gray matter atrophy, attributable to neuronal loss and synaptic reduction (Wu and Gollo 2025). Conversely, immune cell infiltration was markedly elevated in aged animals, especially at 7 dpi, suggesting a shift in the immunomodulatory microenvironment that may actively suppress axonal regeneration.

We further identified remarkable concordance between the temporal expression patterns of EV‐associated genes and pro‐regenerative signatures in neonates, as well as inflammatory and cell‐death related programs in adults. Functional validation showed that inhibition of nSC‐EV secretion impedes scarless repair in neonatal mice, underscoring their essential role in endogenous regeneration. Importantly, tissue‐derived EVs—which better mirror the *in vivo* microenvironment than do EVs derived from *in vitro* ‐ cultured cells—exhibited age‐specific protein profiles reflective of spinal cord mRNA expression patterns. We employed age‐optimized enzymatic digestion protocols to isolate extracellular vesicles from mouse spinal cords across different developmental stages, yielding substantially higher particle numbers per equivalent cell count compared to conventional cell culture‐based methods. Proteomic profiling further confirmed that the observed cargo heterogeneity among nSC‐EVs, aSC‐EVs and oSC‐EVs primarily reflects donor tissue‐intrinsic biological variation. Previous studies have reported marked declines in neuroregenerative capacity in aged mice, linked to dampened expression of neurogenesis‐related genes and reduced neuronal growth potential (Geoffroy et al. 2016; Li et al. 2024; Villeda et al. 2011). Consistent with this, nSC‐EVs demonstrated superior pro‐regenerative activity compared to NSCs‐derived EVs, likely owing to the constrained protein diversity and functionality of *in vitro*‐cultured NSCs. This reinforces the unique regenerative advantage of nSC‐EVs and provides a compelling rationale for their therapeutic use.

Through integrated multi‐omics analyses, we identified Agrin (*Agrn*), L1cam, Epha3/4/5/7/8, and Sema6a/b/d as core signalling molecules enriched in neonatal spinal cord transcriptomes, nSC‐EVs proteomes and snRNA‐seq datasets. While L1cam, Epha and Sema6 families are established regulators of neurite outgrowth and axonal guidance (Gallo et al. 2024; Lee et al. 2024; Lettieri et al. 2023), Agrin (*Agrn*) emerged with heightened specificity in neonatal intercellular communication networks. Previously known for its role in neuromuscular junction formation via MuSK‐Rap1 signalling (Xie et al. 2023) and neonatal heart repair (Bassat et al. 2017; Baehr et al. 2020), we now identify Agrin (*Agrn*) as a pivotal effector of nSC‐EVs‐mediated neurogenesis and spinal cord repair. Single‐cell transcriptomics and immunofluorescence co‐localization further reveal that Agrin (*Agrn*) in nSC‐EVs is predominantly derived from spinal cord microvascular endothelial cells, with functional validation demonstrating that endothelial‐derived Agrin (*Agrn*) is both necessary and sufficient for the neurotrophic activity of the neonatal spinal cord. This expands the functional repertoire of Agrin (*Agrn*) and highlights its therapeutic potential.

We next sought to understand why Agrin is markedly enriched in nSC‐EVs. This enrichment likely reflects two mutually complementary mechanisms. First, neonatal spinal cord tissue exhibits intrinsically high Agrin expression, predominantly in endothelial cells, as revealed by our experiment. Higher cellular protein expression increases molecular availability for EV packaging, thereby elevating protein abundance in secreted EVs (Kalluri and LeBleu 2020; O'Grady et al. 2022). Second, beyond passive enrichment from elevated tissue expression, active selective sorting may further concentrate Agrin into nSC‐EVs. EV biogenesis involves sophisticated cargo selection machinery, which can preferentially recruit specific proteins into intraluminal vesicles regardless of their absolute cytosolic abundance (Xie et al. 2022). Consistent with this notion, our proteomic profiling revealed that nSC‐EVs exhibit a distinct cargo signature rather than merely reflecting the proportional composition of parental cells, with several known EV‐enriched proteins (e.g., TSG101, HSP90) being highly abundant while numerous cytosolic proteins were excluded. Although direct biochemical fractionation would be required to definitively distinguish passive enrichment from active sorting, the convergence of high neonatal tissue expression and EV‐selective packaging mechanisms provides a plausible mechanistic basis for the pronounced Agrin protein enrichment observed in nSC‐EVs.

Despite these advances, several limitations warrant mention. The epigenetic mechanisms governing age‐dependent regenerative‐inflammation balance remain uncharacterized. Temporal multi‐omics integration of SC‐EVs cargo dynamics was not performed, and the therapeutic potential of combining nSC‐EVs administration across injury phases remains unexplored. Furthermore, the cellular provenance of Agrin (*Agrn*) in nSC‐EVs—while strongly implicating spinal cord microvascular endothelial cells—awaits definitive resolution through endothelial‐specific lineage tracing (Cdh5‐Cre or Tek‐Cre). At last, the efficacy and safety of ACPP@nSC‐EVs require validation in non‐human primates. Addressing these questions will be essential for clinical translation.

In summary, this work elucidates that age dictates spinal cord repair competence by modulating the dynamics of regenerative initiation and inflammatory resolution. We systematically decode the regenerative molecular signature of youth *via* multi‐omics profiling and functional studies, and identify nSC‐EVs as a potent, age‐specific mediator of neural repair—a conceptual advance highlighting the functional relevance of tissue‐derived EVs in endogenous regeneration. Moreover, we discovered Agrin (*Agrn*) as a previously unrecognized key effector in spinal cord regeneration, revealing a new dimension of its biological function beyond neuromuscular communication. Therapeutically, we not only establish a targeted delivery platform using ACPP‐functionalized nSC‐EVs to enhance injury‐site accumulation and functional recovery but also provide the first evidence that youth‐derived tissue EVs can rejuvenate adult spinal cord regenerative microenvironments. Our findings open innovative avenues for exploiting innate pro‐regenerative signals and provide a mechanistic and translational foundation for future EV‐based therapies in SCI and other neurological disorders.

## 4 Materials and Methods

### 4.1 Construction of SCI Models in Neonatal‐Adult‐Aged Mice

This study established SCI models in C57BL/6J mice of different ages: neonatal (postnatal day 2, P2), adult (8–10 weeks old), and aged (24‐month‐old). All experiments were conducted in accordance with animal ethical guidelines and approved by the Animal Ethics Committee of the Air Force Medical University of the Chinese People's Liberation Army. Mice were purchased from the Animal Experiment Centre of Air Force Medical University and raised following specific pathogen‐free (SPF) standards. Researchers, trained professionally, strictly adhered to surgical protocols, used sterile gloves and masks and operated with autoclaved instruments. Postoperatively, mice were kept in SPF conditions with clean food and water, and their health was regularly monitored to ensure reliable and reproducible results.

1. Grouping and Anaesthesia Management

Mice were divided into three groups: neonatal, adult and aged. To facilitate post‐SCI urinary assistance, only female mice were chosen for the adult and aged groups. Neonatal mice were anesthetized with isoflurane *via* a small‐animal anaesthesia machine, with respiratory monitoring. Adult and aged mice received intraperitoneal injections of a ketamine (87.5 mg/kg) and xylazine (12.5 mg/kg) mixture. Anaesthesia depth was dynamically adjusted based on respiratory rate and reflexes to ensure pain‐free and stable vital signs.

2. SCI Model Construction Process

Referring to previous studies (Li et al. 2022; Li et al. 2020), we performed a dorsal laminectomy at the T9‐10 vertebrae to fully expose the spinal cord. Then, custom‐made Dumont No. 5 forceps were used to compress the exposed spinal cord for 2 s at a position 5 mm from the forceps' end, inducing complete SCI. After surgery, muscles and skin were sutured layer‐by‐layer, and diclofenac sodium gel was applied for postoperative analgesia.

3. Postoperative Management of Mice
(1) Postoperative Care for Neonatal Mice: (a) Imitating the Natural State: After surgery, a medical cotton swab dipped in maternal feces was gently used to wipe the surgical site of neonatal mice. This aimed to reduce the mother's rejection response, after which the pups were returned to the mother's cage. (b) Thermal and Stress Management: The home cage was placed in a thermostatic incubator, and a recovery box with original bedding was provided overnight to minimize environmental stress. (c) Feeding Monitoring: The postoperative feeding of neonatal mice was closely watched, with a focus on adequate milk intake. (d) Nutritional Support and Euthanasia Criteria: Special breeding‐dedicated feed was supplied to prevent the mother from cannibalizing the pups. Daily weight checks were conducted, and pups with over 10% weight loss were immediately euthanized to prevent unnecessary suffering.
(2) Postoperative Care for Adult and Aged Mice: (a) Recovery and Analgesia: After surgery, adult and aged mice were placed on a thermostatic heating pad until fully awake. Analgesia was administered twice daily for 3 dpi. (b) Bladder Function Management: During the experiment, manual assistance for urination was provided once or twice daily until the study ended. (c) Individual Housing and Observation: Post‐operatively, adult and aged mice were individually housed in an SPF environment. Their behaviour, weight, food/water intake and other physiological indices were closely observed to evaluate the surgical impact on their health.

Through standardized surgery, careful postoperative care, and strict quality control, this study ensured reliable and reproducible results. All procedures were strictly followed by animal ethical guidelines, with various monitoring and management steps taken to minimize animal pain and stress, thus establishing a solid foundation for subsequent research.

### 4.2 Spinal Cord Tissue Perfusion and Immunofluorescence Staining

1. Spinal cord tissue perfusion and processing methods
(1) Perfusion fixation: Experimental mice were administered a lethal dose of anaesthesia and then rapidly perfused *via* the heart sequentially with Phosphate ‐Buffered Saline (PBS) and 4% paraformaldehyde (PFA) to fix the spinal cord tissue.
(2) Shaping and secondary fixation: The mouse spine was placed in a 1 mL pipette tip for shaping and then immersed in PFA for sufficient fixation. Subsequently, the spinal cord tissue was carefully dissected from the spine.
(3) Dehydration treatment: The fixed spinal cord tissue was immersed in a PBS solution containing 30% sucrose at 4°C until the tissue sank to complete the dehydration process.
(4) Tissue embedding and storage: After dehydration, the spinal cord tissue blocks were embedded in OCT embedding medium and stored in a ‐80°C ultra‐low‐temperature freezer.
(5) Section preparation and storage: For sectioning, the frozen tissue blocks were taken out from the ‐80°C freezer and coronal and sagittal sections of 15–20 µm thickness were prepared using a cryostat. The prepared sections were stored at ‐20°C for subsequent experiments.
2. Immunofluorescence staining methods
(1) Section pre‐treatment: Frozen sections were placed in a 37°C oven for 10–20 min to remove moisture. Then, the sections were immersed in PFA for 30 min to stabilize tissue morphology.
(2) Washing treatment: After fixation, the sections were placed in PBS (pH 7.4) and washed three times on a decolorizing shaker for 5 min each time to remove excess fixatives and other impurities from the section surface.
(3) Antigen retrieval: To enhance the accuracy of antigen detection, antigen retrieval was performed by covering the tissue with pepsin and incubating at 37°C for 30 min, followed by rinsing with PBS to expose antigenic epitopes in the tissue and optimize detection conditions.
(4) Secondary washing: After antigen retrieval, the sections were washed again in PBS (pH 7.4) using a decolorizing shaker to ensure no residual retrieval reagents remained on the section surface.
(5) Blocking treatment: A circle was drawn around the tissue with a histological pen, and then the sections were blocked with goat serum for 1 h to prevent nonspecific binding and reduce background interference.
(6) Primary antibody incubation: Primary antibodies from different species were preferentially mixed. The primary antibodies used included mouse anti‐NF‐200 polyclonal antibody (Servicebio, GB12143‐100, 1:500), rabbit anti‐Collagen I antibody (Servicebio, GB11022‐100, 1:200), rat anti‐NeuN antibody (Abcam, ab279297, 1:200), rabbit anti‐CD45 antibody (Servicebio, GB113886‐100, 1:300), rabbit anti‐Cd31 antibody (Servicebio, GB153151‐100, 1:200), mouse anti‐Agrin monoclonal antibody (Santa Cruz, sc‐374117, 1:100), 5‐HT antibody (Servicebio, GB114285, 1:200), Syn‐1 antibody (CST, 5297T, 1:200), MBP antibody (Servicebio, GB12226, 1:500), Map2 antibody (Abcam, ab11267, 1:400), and β‐III‐Tubulin antibody (Abcam, ab78078, 1:200). For BrdU labelling, BrdU (Abcam, ab6326, 1:250) was used to mark β‐III‐Tubulin^+^ cells in double‐labelling experiments. For spinal cord triple immunofluorescence staining, the following rabbit polyclonal antibodies were used sequentially: anti‐S100β (Servicebio, GB11359, 1:2000), anti‐MBP (Servicebio, GB11226, 1:100), and anti‐OLIG2 (Servicebio, GB15766, 1:200). The mixed primary antibody solution was applied to the section surface, which was then placed in a humidified chamber and incubated overnight at 4°C. When labelling homologous species antibodies (such as two rabbit‐sourced primary antibodies), sequential incubation was used.
(7) Secondary antibody incubation: The sections were thoroughly washed before secondary antibody incubation. The secondary antibodies used included Alexa Fluor 488 and 594‐labeled ones, which needed to match the species of the primary antibodies. The sections were incubated at room temperature in the dark for 60 min to allow specific binding of the secondary antibodies to the primary antibodies.
(8) In mounting and image acquisition/analysis, the sections were mounted with an antifade mounting medium containing DAPI, and then imaged and analyzed using a confocal laser scanning microscope.
(9) Fiji 2.14.0 (ImageJ2 core) was used for immunofluorescence analysis. The immunofluorescence images were imported into the software for analysis.

### 4.3 Bulk RNA Sequencing

1. Sampling and sequencing library construction process: (1) Tissue: This study employed bulk RNA sequencing to investigate the transcriptomic alterations in spinal cord tissues of mice across different age groups. This encompassed uninjured spinal cords from neonatal, adult and aged mice, along with spinal cords harvested from neonatal and adult mice at 1, 3 and 7 days following SCI. All tissue samples were procured from the T9‐10 spinal segment. For the injured cohort, the collected samples comprised spinal tissue situated within 1 mm rostrally and caudally to the injury epicenter. To guarantee the scientific rigor and reliability of the findings, stringent experimental protocols and comprehensive quality control measures were meticulously implemented throughout the procedure. (2) Cells: Initially, nSC‐EVs and aSC‐EVs were incorporated into the prepared NSCs culture system. On day 3 post‐treatment, the NSCs were gently washed with PBS to eliminate impurities present in the culture medium. Subsequently, the cells were harvested byTrypLE Express enzyme digestion. The harvested NSCs were then subjected to counting *via* an automated cell counter. Cell viability was assessed employing the trypan blue exclusion assay to ensure the collected cells met the experimental viability criteria. Total RNA was extracted from the NSCs using a TRIzol‐based RNA extraction reagent. Particular emphasis was placed on preventing RNA degradation and avoiding exogenous RNA contamination during the extraction process.
2. RNA Extraction: All procedures were performed in strict accordance with the manufacturers' protocols. RNA quality was rigorously assessed using an Agilent 2100 Bioanalyzer. The RNA purity (A260/A280 ratio) was maintained between 1.8 and 2.2, and the RNA integrity number was kept at or above 7.0.
3. Library preparation and sequencing: Library preparation was performed using the Illumina TruSeq Stranded mRNA LT Library Prep Kit. RNA samples were first purified with polyadenylation magnetic beads, and then the library was constructed following the standard protocol. To ensure library quality, cluster generation was carried out using the cBot Cluster Generation System, and high‐throughput sequencing was performed on the Illumina NovaSeq 6000 sequencing platform in PE150 mode. Prior to sequencing, the library concentration, insert fragment size, and uniformity were strictly tested and the insert fragment size distribution was uniform.
4. Data Processing Pipeline: The sequencing data were processed through several key steps. First, the adapter sequences were removed, followed by the elimination of low‐quality reads and repetitive sequences. Subsequently, the clean reads were aligned to the mouse genome reference mm10 using Hisat2, and gene expression levels were quantified based on count values. To ensure the reliability and consistency of the data, PCA was employed to detect outliers among samples, and data analysis was performed using various packages in R software.

In this study, standardized experimental procedures and stringent quality control measures were implemented to ensure the accuracy and reliability of each step.

### 4.4 Single‐Cell Nuclear RNA Sequencing

In this study, the snRNA‐seq samples of uninjured, 3 dpi and 7 dpi from neonatal and adult mice, as well as the uninjured and 3 dpi samples from aged mice, were analyzed by Shanghai Oei Biomedical Technology Co., Ltd. The 7 dpi samples of the aged group were obtained from the GSM7474498, GSM7474499 and GSM7474500 samples in the GSE234774 dataset (O'Shea et al. 2024; Skinnider et al. 2024). The specific method is as follows:

(1) Sample Collection and Processing

Mice were euthanized by lethal anaesthesia, after which the target tissues were rapidly excised and immediately immersed in liquid nitrogen to ensure RNA integrity. The snap‐frozen tissue samples were transported to the facility on dry ice for subsequent processing. Prior to formal sample processing, a portion of each tissue sample was sectioned for RNA quality assessment. RNA quality control was performed using the Agilent RNA 6000 Nano Kit, with only samples exhibiting an RNA Integrity Number (RIN) of ≥7.0 being retained for further experimentation to ensure reliability.

For formal processing, the liquid nitrogen‐frozen spinal cord tissues were briefly rinsed in PBS to eliminate potential contaminants and residual liquids. The tissues were then sectioned and carefully placed into cryovials containing 1 mL of tissue RNA preservation solution, ensuring complete submersion to maintain RNA stability and integrity.

Nuclear extraction was subsequently performed. The tissue pieces were placed on ice, and pre‐chilled NP40 Lysis Buffer was added. Using surgical scissors, the tissues were thoroughly minced on ice to facilitate sufficient contact between tissue cells and the lysis solution, thereby promoting cell lysis and releasing the nuclei. The minced tissue mixture was then filtered through a 40 µm cell strainer to allow nuclei to pass through while intercepting larger tissue fragments and other impurities. The filtrate containing the nuclei was centrifuged at 1000 g, 4°C, for 5 min. After centrifugation, the supernatant was discarded, and the nuclei were resuspended in PBS and subjected to another round of centrifugation and washing, a process repeated 2–3 times to completely remove residual lysis solution and other impurities, ensuring nuclear purity and activity.

The quality of the nuclei was assessed to ensure the absence of clumps or particulate impurities with a diameter exceeding 40 µm, a fragmentation rate of less than 20%, an aggregation rate of less than 10% and a total count of over 10^5^ nuclei.

(2) Sequencing Library Construction and Initial Data Processing

Sequencing libraries for the isolated cell nuclei samples described above were constructed using the 10x Genomics Chromium platform.snRNA‐seq reads were aligned to the Ensembl file (GRCm38.101) of the mouse genome using CellRanger (10X Genomics, v.4.0.0) to obtain unique molecular identifier (UMI) count matrices. Data preprocessing was carried out using the Seurat software package. Initially, cells with feature gene counts between 200 and 6000 were selected to ensure adequate gene expression information without over‐expression. Subsequently, doublets were removed using DoubletFinder. Finally, cells with low expression levels of mitochondrial and ribosomal genes were screened, requiring mitochondrial gene expression to be below 15% and ribosomal gene expression below 30% to guarantee cell quality. After quality control, the top 2000 highly variable genes were identified using the FindVariableGenes function. Dimensionality reduction was then performed using PCA,, Batch effects between the in‐house and GSE234774 datasets were corrected using Harmony. The Harmony ‐ corrected embeddings were than used for UMAP visualization. Next, DEGs were detected using the FindAllMarkers function and visualized with the V lnPlot and FeaturePlot functions. Cell‐type identification was conducted using the SingleR software by calculating the correlation of cell expression profiles with public reference datasets and manually annotating cell types based on previous literature. The entire experimental process strictly adhered to standardized operating procedures to ensure data reliability and analytical accuracy.

### 4.5 Omics Data Analysis and Functional Interpretation

To thoroughly explore the biological significance underlying the sequencing data, this study primarily utilized the R programming language and the following analytical approaches:

(1) Differential analysis: The limma package was employed to identify DEGs by comparing gene expression profiles across distinct groups. Rigorous filtering criteria were applied, requiring a log_2_ fold change absolute value (log_2_|Fold Change|, hereafter |log FC|) ≥ 1 and *P*‐value < 0.05. Volcano plots were generated to visually represent the DEGs.
(2) Functional clustering analysis: The expression patterns of genes were cluster analyzed *via* an unsupervised clustering algorithm, the mfuzz algorithm. The functional module classification of genes and their expression patterns was presented in the form of heatmaps, etc., further clarifying the changing trends and biological significance of each functional module across different groups.
(3) Gene‐set function‐based enrichment analysis: We conducted Gene Ontology (GO) analysis on DEGs and gene sets in clustered modules using R. We explored their roles in biological processes, cellular components, and molecular functions. We also used the Kyoto Encyclopedia of Genes and Genomes (KEGG) database for pathway enrichment analysis. The workflow was as follows: First, we used the bitr function in the clusterProfiler package to convert gene symbols to Entrez IDs. Then, we used the enrichGO function (with org. Mm.eg.db as the species annotation database) for GO enrichment analysis and the enrichKEGG function for pathway enrichment analysis. Finally, we used the ggplot2 package to optimize visualizations, presenting the results as bubble charts, radar charts, bar charts, etc.
(4) Gene Set Enrichment Analysis (GSEA): Since the aforementioned GO and KEGG enrichment analyses primarily focus on DEGs and neglect genes with statistically significant expression differences but absolute log FC values not meeting threshold criteria, GSEA was employed to comprehensively assess the enrichment of DEGs in specific biological processes and pathways, thereby uncovering their potential biological functions. Essential R packages, including clusterProfiler, org.Mm.eg.db, and enrichplot, were loaded. Subsequently, the gseGO function was utilized to perform GO enrichment analysis on gene expression data, with GO terms having *P*‐values below 0.05 and absolute enrichment scores exceeding 0.25 being selected. Then, the gseKEGG function was applied to conduct KEGG pathway enrichment analysis on mouse gene expression data, with results sorted in descending order of enrichment scores using the same selection criteria as for GO analysis. Ultimately, visualization was achieved using packages such as aPEAR and GseaVis.
(5) Single‐sample Gene Set Enrichment Analysis (ssGSEA): In this study, we established a gene set extraction framework by extracting gene set data of mice from the org.Mm.eg.db database. First, we utilized the get_GO_data function to obtain GO data and saved it as the GO_DATA_mmu.RData file. Subsequently, we designed two functions: findGO and getGO. FindGO can find relevant GO pathways based on keywords or gene names, and getGO can extract the corresponding gene sets based on specified GO IDs. These methods enhanced the efficiency of gene set extraction and provided a convenient tool for subsequent ssGSEA analysis. Finally, based on the GSVA package, we quantified the activity scores of specific gene sets in each sample and further explored the differential trends of pathway activity and their biological significance among different groups.
(6) Area Under the Curve of cell expression (AUCell): The activity of gene sets in snRNA‐seq gene expression data was evaluated using the AUCell analysis method. Initially, snRNA‐seq gene expression data were processed into a sparse matrix format by loading relevant R packages (such as AUCell and Seurat). Subsequently, the AUCell_buildRankings function from the AUCell package was employed to generate a gene expression ranking matrix, and the AUCell_calcAUC function was utilized to calculate the AUC values of each cell for the gene sets. Then, AUCell_getAUC was applied to extract each cell's AUC scores, representing the activity of the corresponding gene sets in the cells. Finally, the activity patterns of gene sets were visualized through violin plots, box plots and UMAP plots. Subsequent analyses could compare gene set activity scores across different sample populations and cell clusters.
(7) Single‐cell CellChat communication analysis: Based on snRNA‐seq data, we utilized the CellChat tool to construct intercellular communication networks and decipher the transmission patterns of key signalling pathways among diverse cell types, as well as their potential roles in pathological processes. Given that prior studies predominantly relied on scRNA‐seq, which tends to capture more immune cells due to their robustness during enzymatic dissociation while neurons are frequently lost, we opted for snRNA‐seq as it is less biased toward immune cells and better preserves neuronal populations. Initially, we employed the R package CellChat to load snRNA‐seq gene expression data and created a CellChat object *via* the createCellChat function. Subsequently, the data were processed, and overexpressed ligands and receptors in each cell population were identified using the processData and identifyOverExpressedGenes functions. Then, the communication probability between cells was calculated using the computeCommunProb function, and the communication pathways were evaluated by applying the computeCommunProbPathway function. Afterward, visualization functions such as netVisual_aggregate were used to depict the intercellular communication networks and signal transduction pathways. Finally, through atlas analysis and statistical tests, we gained an in‐depth understanding of the communication mechanisms among different cell populations.

### 4.6 Isolation and Extraction of EVs From Spinal Cord Tissues, NSCs and Neurons

In this study, we performed the isolation, purification and identification of spinal cord‐derived and cell‐derived EVs. The specific experimental methods are as follows:

(1) Pre‐processing of SC‐EVs before purification

We established an SC‐EVs isolation and purification protocol adapted to the characteristics of spinal cord tissue by refining the method from the literature (Lou et al. 2023). The specific operational processes are as follows.

(2) Experimental animal processing and tissue pretreatment: C57BL/6J mice (age ranging from the neonatal period to adulthood and old age) were sacrificed by cervical dislocation following isoflurane anaesthesia, with strict adherence to the principles of laboratory animal ethics throughout the procedure. The spinal cord tissues were rapidly excised and washed three times with pre‐cooled sterile PBS, then finely trimmed into 1 mm^3^ tissue blocks using sterile micro‐scissors on ice.
(3) Enzymatic Digestion System Construction: (1) Tissue enzymatic digestion. In response to the unique glial fiber network and basement membrane complex structure of spinal cord tissue, an optimized enzymatic digestion protocol was established. A mixture of 0.25% trypsin and 0.1% collagenase IV (volume ratio 1:1) was prepared and digestion was carried out in a 37°C constant‐temperature shaker. The digestion time was adjusted according to the developmental stage of the animal: 1 h for neonatal mice and 2 h for adult and aged mice. These durations were empirically optimized to achieve saturated EV release based on tissue biomechanical properties and extracellular matrix (ECM) density across developmental stages (Crescitelli et al. 2021; Lou et al. 2023). Preliminary experiments confirmed that neonatal spinal cord tissues, characterized by loose ECM enriched in hyaluronic acid with dispersed collagen fiber arrangement, reached maximal EV yield at 1 h with no further increase upon extension to 2–3 h. Conversely, adult and aged tissues, exhibiting denser ECM architecture, heightened collagen cross‐linking, and progressive accumulation of matrix metalloproteinase inhibitors that impede enzymatic penetration, required 2 h to achieve comparable yield saturation; prolongation to 3 h failed to elevate EV output. Furthermore, Proteomic analysis of EVs isolated after 1 h, 2 h and 3 h digestion revealed highly overlapping proteomic profiles with no systematic up‐ or down‐regulation of protein abundances, confirming that differential digestion duration does not alter intrinsic EV cargo composition. To eliminate interference from exogenous vesicles, Exo‐Free FBS‐containing medium was used throughout the entire process. Gentle pipetting was performed every 5–10 min using a blunt‐tip pipette. After digestion, the mixture was sequentially filtered through 100 µm and 40 µm pore‐size filters to remove undigested tissue fragments. The filtrate was stored at low temperature for subsequent use. (2) The primary NSCs and neuron culture media derived from the spinal cord were collected using the Exo‐Free FBS medium throughout the process. (3) Impurities were removed by differential centrifugation. A differential centrifugation strategy was employed to sequentially remove impurities from enzymolysis products and cell supernatants. Initially, centrifugation was performed at 300 × g for 10 min at 4°C, with supernatants collected and incompletely dissociated tissue debris discarded. Subsequent steps included centrifugation at 2000 × g for 30 min to clear apoptotic bodies and free organelles larger than 1 µm in diameter, followed by centrifugation at 4000 × g for 60 min to remove mitochondrial and endoplasmic reticulum fragments. Ultracentrifugation was then conducted at 120,000 × g for 70 min at 4°C. The final purified EV pellets were resuspended in sterile PBS, washed, aliquoted, and stored at ‐80°C for later use.

### 4.7 Behavioural Assessment of Motor Function in Mice

In this study, we used multiple methods to assess motor function recovery in mice following SCI.

(1) Basso Mouse Scale (BMS) scoring: This study used the BMS scoring to quantitatively evaluate hindlimb motor function (Basso et al. 2006). This method uses a 9‐point scale in a quiet, bright, open environment to comprehensively assess hindlimb joint mobility, gait coordination, weight‐bearing, and range of motion, as higher scores indicate better motor function recovery. To ensure objective and reliable results, all experiments were performed by three trained observers under a double‐blind design, where they were unaware of the experimental grouping to minimize subjective bias. Observers independently scored based on specific hindlimb motor performances. After testing, they fully discussed and reached a consensus on the final scores. All experimental data were recorded in real‐time with regular quality checks to ensure accuracy and consistency.
(2) Catwalk gait analysis: We assessed motor function recovery using the Catwalk analysis platform (Timotius et al. 2023). Mice walked through a dark glass walkway with side panels ensuring single‐mouse passage. As they walked, pressure from their limbs made the glass surface emit green light, and a high‐speed camera recorded the signals. After the mice passed, CatWalk^TM^ XT 8.1 software analyzed gait parameters and motility to evaluate hindlimb motor function. This method allows quantitative observation of motor function changes and provides accurate behavioural recovery data.
(3) Electrophysiological Analysis: To evaluate the functional state of somatosensory and motor signal conduction in experimental animals, electrophysiological tests were conducted at 42 dpi. Before the experiment, experimental animals were anesthetized using the aforementioned anaesthesia method, and then the right sciatic nerve was exposed. Subsequently, stimulating electrodes were precisely inserted into the motor cortex area and recording electrodes were placed on the sciatic nerve. During the experiment, standardized square‐wave pulse stimulation was applied, and motor evoked potential (MEP) signals were continuously acquired. All signals were digitally processed and stored.

For the collected electrophysiological data, the waveform characteristics, amplitude and latency of the evoked potentials were analyzed to assess the functional state of somatosensory and motor signal conduction. The entire experimental process was strictly followed by the requirements of animal ethics and was operated by experienced researchers to ensure the accuracy and reliability of the experimental data.

### 4.8 Statistical Analysis

Statistical differences were analyzed using R (version 4.2.0) or GraphPad Prism (version 9.2.0). Animal data are presented as counts (*n*) and percentages. Normality was assessed using the Shapiro‐Wilk test and Q‐Q plots. All experiments were performed using simple randomization, in which animals were randomly allocated to each group. Prior to analysis, data were tested for normality and homoscedasticity. For normally distributed data, comparisons between two groups were performed using Student's *t*‐test, whereas comparisons among more than two groups were conducted using one‐way ANOVA followed by Bonferroni's or Tukey's multiple comparisons post hoc test. For non‐normally distributed data, two‐group comparisons were analyzed using the Mann–Whitney U test, and comparisons among three or more groups were performed using the Kruskal–Wallis test followed by Dunn's multiple comparisons test.

Statistical significance was defined as *P* < 0.05. Further statistical details are provided in the figure legends.

## Author Contributions

**Yongfeng Zhang**: investigation, visualization, writing – original draft, writing – review and editing, conceptualization, methodology. **Qi Liu**: methodology, writing – review and editing, formal analysis, software, investigation. **Beibei Yu**: methodology, writing – review and editing. **Borui Xue**: methodology, software, writing – review and editing. **Shijie Yang**: investigation. **Teng Ma**: investigation, funding acquisition. **Lingli Guo**: investigation. **Haining Wu**: investigation. **Xue Gao**: visualization. **Shengyou Li**: visualization. **Yujie Yang**: visualization. **Yiming Hao**: validation. **Ziqing Zhu**: validation. **Meijia Su**: software. **Hongyu Li**: software. **Shuanshuan Fan**: software. **Jiaqi Wang**: software. **Huangtao Chen**: software. **Longhui Fu**: validation. **Anhui Qin**: data curation. **Zhenguo Wang**: data curation. **Shihao Nie**: writing – review and editing. **Mingze Qin**: writing – review and editing. **Xinxin Bai**: writing – review and editing. **Guang Kong**: writing – review and editing. **Rui Zhao**: writing – review and editing. **Dong Lei**: writing – review and editing. **Yue Yu**: writing – review and editing. **Junxiong Ma**: investigation, supervision. **Shouping Gong**: conceptualization, supervision. **Bing Xia**: conceptualization, writing – review and editing, supervision, funding acquisition. **Jinghui Huang**: conceptualization, writing – review and editing, writing – original draft, supervision, funding acquisition.

## Funding

This work was supported by the National Key R&D Program of China grants 2024YFA1802502(J.H.), the National Natural Science Foundation of China grants 82525039(J.H.), 82472418(B.X.), 82372404(J.H.), 82430077(Z.L.), 82502883(S.L.) and 82472417(T.M.).

## Conflicts of Interest

The authors declare no conflicts of interest.

## Supporting information




**Supporting file**: jev270371‐sup‐0001‐SuppMat.docx

**Supporting file**: jev270371‐sup‐0002‐DataS1.xlsx

**Supporting file**: jev270371‐sup‐0003‐DataS2.xlsx

**Supporting file**: jev270371‐sup‐0004‐DataS3.xlsx

**Supporting file**: jev270371‐sup‐0005‐DataS4.xlsx

**Supporting file**: jev270371‐sup‐0006‐DataS5.xlsx

## Data Availability

The data that supports the findings of this study are available in the supplementary material of this article. Additional materials or datasets can be obtained from the corresponding author upon reasonable request.
