# Supplementary material for: Agrin‐Enriched Neonatal Spinal Cord‐Derived Extracellular Vesicles Bypass Age Barriers to Organize Spinal Cord Repair
Source: J Extracell Vesicles. 2026 Oct 10;15(10):e70371. doi: 10.1002/jev2.70371 (PMC13653649; doi:10.1002/jev2.70371)
Supplement: Supplementary file 1 [file JEV2-15-e70371-s005.docx]

Supplementary Materials for

**Agrin-enriched neonatal spinal cord-derived extracellular vesicles bypass age barriers to organize spinal cord repair.**

Yongfeng Zhang *et al.*

*Corresponding author. Email: huangjh@fmmu.edu.cn (J.H.); xiabing8807@fmmu.edu.cn (B.X.); shpingg@126.com (S.G.); cliffmjx@163.com (J.M.).

**This PDF file includes:**

Supplementary Materials and Methods

Figs. S1 to S19

Tables S1 to S3

Data S1 to S5

**Supplementary Materials and Methods**

**1 | TEM identification of multivesicular bodies**

1) Observation of Multivesicular Bodies: Spinal cord tissue was harvested and trimmed into 1 mm³ tissue blocks. The samples were fixed in 2.5% glutaraldehyde at 4°C for 24 h, followed by washing with PBS. Subsequently, the samples were fixed in 1% osmium tetroxide at 4°C for 2 h. After dehydration through a graded acetone series, the samples were embedded in epoxy resin at 37°C for 12 h and then polymerized in an oven at 65°C for 48 h. Ultrathin sections (60-90 nm) were prepared, stained with uranyl acetate and lead citrate, and observed under a TEM to examine the ultrastructure of multivesicular bodies.

2) EV observation procedure: First, place the grid with its clean side facing upward and add a drop of EVs suspension onto it. After allowing 10 min for adsorption, carefully remove excess liquid. Transfer the grid to glutaraldehyde fixative for a 5 min immersion. Following blotting, rinse it with deionized water 10 times (each rinse lasting for 2 min), blotting between each rinse. Then immerse the grid in uranyl acetate stain for 10 min. After blotting again, transfer it to methylcellulose solution for 5 min. After sufficient drying (≥ 30 min), the grid is ready for TEM observation and image recording.

**2 | Isolation, identification, and culture of primary NSCs**

1) Experimental animals: In this study, embryos of C57BL/6J mice at embryonic day 14 (E14) were chosen as the experimental materials.

2) Isolation and culture of spinal cord NSCs: Within a biosafety cabinet, the embryos were washed with pre-chilled PBS. Under a stereomicroscope, the spinal cord tissues of mouse embryos were accurately dissected and cut into tissue chunks smaller than 1 mm³. The tissue chunks were placed in a digestion solution containing 0.1% collagenase type IV and digested at 37°C for 10 min. After digestion, cells were dispersed by gently trituration with a fire-polished glass pipette and then filtered through a 40 μm cell strainer to obtain a single-cell suspension.

3) Cell viability assessment and culture Seeding: Cell viability was assessed using trypan blue staining to ensure a viability rate of over 95%. Cells were suspended in NSC complete culture medium (Neurobasal medium supplemented with B27, growth factors, etc.) and seeded in culture dishes.

4) Culturing and passaging: When the neurospheres reached a diameter of 150-200 μm and confluence reached 80%, they were dissociated using TrypLE Express enzyme at 37℃ for 3 min, followed by gentle mechanical dissociation with a fire-polished pipette. The cells were then passaged and expanded until the third passage (P3), after which the supernatant was collected for subsequent experiments.

5) NSC characterization: NSCs were identified *via* cell immunofluorescence staining. Rabbit anti-Nestin (Proteintech, 19483-1-AP,1:100) and mouse anti-Sox2 (Proteintech, 66411-1-Ig,1:200) were used as primary antibodies and incubated overnight at 4℃. Then, cells were incubated with Alexa Fluor 488/594-conjugated secondary antibodies for 1 h at room temperature in the dark. Images were captured using a confocal microscope. Image-J was used to analyze the positive expression rate. Results showed that the positive rates of Nestin and Sox2 both exceeded 90%, confirming the NSCs characteristics of the obtained cells.

**3 | Isolation, identification, and culture of primary spinal cord neurons**

1) Experimental material pretreatment: The pretreatment of culture vessels employed PLL coating technology. 24 h prior to cell seeding, 100 μg/mL PLL solution was added to the culture dishes, which were then incubated at 37°C for 12 h. After incubation, the coating solution was removed and the dishes were washed three times with PBS, followed by drying at room temperature for subsequent use. Two types of neuron culture media were utilized in this study: the seeding medium was prepared based on high-glucose DMEM, supplemented with 10% FBS, 5% horse serum, and 1% penicillin-streptomycin solution; the maintenance medium consisted of Neurobasal medium enriched with 2% B27 supplement, 2 mmol/L L-glutamine, and 1% penicillin-streptomycin solution.

2) Isolation of embryonic spinal cord tissue: Pregnant C57BL/6J mice at 14-16 days of gestation were selected. After anesthesia, the abdominal skin was routinely disinfected. Under sterile conditions, the abdomen was opened to remove the uterus intact, which was then placed in pre-cooled HBSS. Under a stereomicroscope, the embryos were isolated one by one, with the placenta and accessory tissues removed. The vertebral canal was incised along the midline of the spine, and the spinal cord tissue was dissected out. Throughout the procedure, the tissue was kept moist, and the isolated spinal cords were immediately placed in ice-cold HBSS. Spinal cords were then processed in batches of 10 embryos.

3) Primary Neuronal Culturing: First, spinal cord tissues were sectioned into approximately 1 mm³ fragments. Subsequently, these tissue fragments were divided into two groups and subjected to digestion in Accutase enzyme solution at 37℃. During this process, the test tubes were gently shaken every 5 min to ensure adequate mixing of the contents. Upon completion of the digestion, an equal volume of serum-containing culture medium was added to neutralize the enzymatic activity. Then, cells were dispersed by aspirating and dispensing the solution approximately 20 times with a 1 mL pipette tip gently. After a 1-min settling period, the supernatant containing free spinal cord neurons was collected, while the undigested tissue residues at the bottom were subjected to a second round of digestion with Accutase enzyme solution at 37℃, with the same shaking regimen applied every 5 min. Following this, the enzymatic activity was once again neutralized with serum-containing culture medium, and the cells were gently triturated 20 times.

The collected cell suspensions were then filtered through a 70 μm cell strainer to eliminate debris. Next, the filtrate underwent centrifugation at 1000 rpm for 5 min. The supernatant was discarded, and an appropriate volume of serum-containing culture medium was added. The cells were gently resuspended by mixing several times with a 1 mL pipette tip.

4) Trypan blue staining revealed that the cell viability was no less than 80%.

5) Seeding, purification, and medium change: Cell suspensions were seeded into PLL coated 6 -well culture plates at a density of 1.5×10⁶ cells per well, with 3 mL of cell suspension added to each well. After 1 d of culture, the original culture medium was completely replaced with serum-free neurobasal medium suitable for neurons. After an additional 2 d of culture, cytosine arabinoside was added to the culture medium to a final concentration of 5 μmol/L and incubated for 24 h. Subsequently, the culture medium was again completely replaced with serum-free neurobasal medium. Thereafter, half of the medium was changed every 3 d. On day 7, the culture supernatant was collected and EVs were isolated from it.

6) Neuronal-specific identification: Indirect immunofluorescence was used to detect the expression of β-III-Tubulin protein. Cells at day 3 of culture were subjected to immunofluorescence staining. They were sequentially incubated with mouse anti-β-III-Tubulin antibody (Abcam, ab78078, diluted at 1:200) and Alexa Fluor 488-labeled goat anti-mouse secondary antibody (1:200), with DAPI used for nuclear staining.

**4 | Multidimensional identification and characterization of EVs derived from tissues and cells**

Various technical approaches were utilized in this study to conduct morphological observation, concentration quantification, and marker detection of the isolated EVs, so as to ensure their purity and specificity.

1)Morphology: TEM was used to identify the morphological characteristics of EVs according to the aforementioned method, with careful observation of their diameter and typical morphology.

2) Size analysis: NTA was used to determine the concentration and size distribution of EVs in the samples. EVs were diluted with sterile PBS to a concentration suitable for detection and then analyzed using the ZetaView™ NTA system. The detection parameters were set as follows: temperature at 25°C and laser wavelength at 520 nm. Finally, the concentration and average size of EVs in the samples were obtained.

3) Canonical markers were assessed by Western blotting: Hsp90 and TSG101 served as positive markers, whereas Calnexin—which is scarce in EVs yet abundant in cell and tissue lysates—was used as a negative control. Experimental details are provided in the Western blotting section.

4) We employed the ExoView platform to achieve multiparametric characterization of EVs. Following standardized protocols, EVs were loaded onto the ExoView chip (EV-TM-FLEX-CAR). Fluorescently labeled antibodies targeting CD9, CD81, and CD63 were utilized for the specific capture and detection of surface markers on EVs. The ExoView system, leveraging high-resolution imaging technology, performed quantitative analysis of marker expression (*Breitwieser et al. 2022*).

**5 | miRNA sequencing and integrative target prediction**

Total RNA extracted from SC-EVs was subjected to miRNA library construction and high-throughput sequencing by Biomarker Technologies (Beijing, China). Raw sequencing reads underwent standard preprocessing, including adapter trimming and quality filtering, followed by alignment to the mouse reference genome (mm10) and annotation against the miRBase database. Differential expression analysis was performed using the DESeq2 package in R. Downstream target gene prediction was conducted through the multiMiR database.

**6 | Protein extraction and Western blotting**

In this study, quantitative analysis of target proteins was performed using standard protein extraction and Western blotting. The main reagents used in the experiment included RIPA lysis buffer (containing protease and phosphatase inhibitors), SDS-PAGE gel preparation reagents, 5×loading buffer, PVDF membranes, BCA protein quantification reagent kit, and the corresponding antibody reagents.

1) Preparation of experimental materials: The cell cultures were prepared and routinely cultured in advance before subsequent processing. The EV samples were collected and washed with PBS prior to further processing.

2) Protein extraction from cells and EVs: The cells and EVs were prepared for protein extraction. Cell samples were washed with PBS and lysed with RIPA lysis buffer. After sonication on ice, the lysates were centrifuged at 12,000 × g for 15 min at 4°C, and the supernatant was collected. For EVs, after being resuspended in PBS, they were lysed with RIPA buffer containing inhibitors. Following incubation on ice, the EV lysates were centrifuged under the same conditions to obtain the supernatant as the protein extract.

3) For protein quantification and Western blotting, the BCA assay kit was used to measure protein concentrations at 562 nm, and all samples were adjusted to a uniform concentration. The specific steps for Western blotting are as follows:

(1) Preparation of Gels and Electrophoresis: Prepare a 5% stacking gel and separating gels of appropriate concentration according to the target protein molecular weight (6–8% for proteins >150 kDa, such as Ki67 and Agrin; 10% for proteins of 30–150 kDa). Mix 20 μg of protein samples with 5×SDS-PAGE loading buffer (final 1×, containing a reducing agent) and denature at 95 °C for 5 min. Electrophorese at constant voltage: 80 V in the stacking gel, then 120 V after the dye front enters the separating gel.

(2) Transfer: After electrophoresis, transfer proteins to PVDF membranes using the wet transfer method, maintaining an ice bath environment throughout the transfer process.

(3) Immunoblotting: Block PVDF membranes with 5% skim milk for 1 h. Incubate with specific primary antibodies: rabbit anti-Calnexin antibody (Proteintech, 10427-2-AP, 1:1000), rabbit anti-TSG101 antibody (Abcam, ab125011, 1:1000), rabbit anti-Hsp90 antibody (CST, 4874S, 1:1000), mouse anti-KI67 antibody (Thermo Fisher Scientific, MA5-15484, 1:1000), rabbit anti-β-Catenin antibody (WanleiBio, WL0962a, 1:500), rabbit anti-GSK3β antibody (WanleiBio, WL01456, 1:500), rabbit anti-p-GSK3β antibody (WanleiBio, WL03683, 1:500), mouse anti-Agrin monoclonal antibody (Santa Cruz, sc-374117, 1:500), and rabbit anti-GAPDH antibody (Servicebio, GB15004-100, 1:2000). Incubate with the primary antibody overnight at 4°C, followed by incubation with the secondary antibody.

After primary antibody incubation, wash the membrane three times with TBST for 15 min each. Then incubate with HRP-conjugated secondary antibodies for 1 h at room temperature. Capture images using ECL chemiluminescent imaging system and finally analyze the band gray values.

**7 | Hematoxylin-Eosin (H&E) and Nissl staining**

1) H&E Staining

(1) Bake paraffin-embedded sections (10 μm) at 60°C for 4 h to ensure tissue adhesion.

(2) Deparaffinize in xylene (2 × 10 min), rehydrate through graded ethanol (100%, 95%, 90%, 80%, and 70%, 5 min each), and rinse in distilled water for 1 min.

(3) Stain with hematoxylin for 2 min, differentiate in 1% hydrochloric acid in ethanol, rinse under running water until nuclei turn blue, and counterstain with 0.5% eosin for 1 min.

(4) Dehydrate through graded ethanol (70–100%), clear in xylene (2 × 5 min), and mount with neutral balsam.

(5) Image sections using a Vectra Polaris multispectral imaging system (Akoya Biosciences).

2) Nissl Staining:

(1) All procedures were performed strictly in accordance with the instructions of the Beyotime kit.

(2) Section processing: Sections were sequentially treated with xylene, anhydrous ethanol, 95% alcohol, 80% alcohol, 70% alcohol, and distilled water, with the timing referenced to the H&E staining procedure.

(3) Staining: Stain with 1% cresyl violet for 30 min.

(4) Post-treatment: Rinse with distilled water, differentiate in 70% alcohol for 2 min, then dehydrate sequentially in 70%, 80%, and 95% alcohol for 2 min each, followed by two 5-min treatments with anhydrous ethanol and two 10-min treatments with xylene.

(5) Mounting: Mount with DPX. Nissl bodies appear purple and cell nuclei appear pale purple.

(6) Image acquisition and analysis: Images of spinal cord neurons were captured using an inverted microscope, and intact neurons in six non-consecutive fields of view per section were quantitatively analyzed using Fiji 2.14.0.

**8 | Cellular uptake assay of EVs**

1) Isolation and labeling of SC-EVs

(1) Collection and resuspension of SC-EVs: After collecting SC-EVs, resuspend them in PBS to prepare a suspension of appropriate concentration for subsequent staining.

(2) Dye Preparation: Take an appropriate amount of PKH26 dye and dilute it according to the kit instructions to prepare a working concentration dye solution.

(3) Labeling Reaction: Mix the resuspended SC-EVs with the diluted PKH26 dye solution in a certain proportion and gently vortex to mix thoroughly. Then incubate at room temperature in the dark for 5 min to ensure adequate labeling.

(4) After incubation, add an appropriate amount of stop solution (DF-12 medium with 10% FBS) to terminate the labeling reaction.

2) Isolation of PKH26-labeled SC-EVs

(1) Transfer the suspension of labeled SC-EVs to a centrifuge tube and ultracentrifuge at 100,000 × g for 2 h at 4°C to remove free PKH26 dye and other impurities, obtaining purified labeled SC-EVs.

(2) After centrifugation, resuspend the precipitated SC-EVs in PBS for subsequent experiments.

3) Coculture of SC-EVs and neuronal cells

For the co-culture assay, neuronal cells were incubated with PKH26-labeled SC-EVs at the **working concentration** of **10 μg/mL** for 24 h at 37°C. Then follow the immunofluorescence staining steps in aforementioned sections: add the primary antibody against β-III-Tubulin (Abcam, ab78078, diluted at 1:200). Finally, observe the neuronal cells under a fluorescence microscope, monitoring the fluorescence of β-III-Tubulin (green), nuclei (blue), and PKH26-labeled SC-EVs (red) through different channels to evaluate the uptake efficiency and distribution of SC-EVs.

**9 | Method for using Wnt/β-catenin pathway inhibitors**

To investigate the regulatory mechanism of nSC-EVs on NSCs, this study employed the small-molecule inhibitor XAV939 to selectively inhibit the Wnt/β-catenin signaling pathway (*Pundkar et al. 2023*).

1) Grouping and treatment of NSCs:

(1) Vehicle group: Treated with an equal volume of DMSO only.

(2) nSC-EVs group: NSCs are treated with an equal volume of DMSO and exposed to nSC-EVs.

(3) XAV939 group: NSCs are treated with 10 μM XAV939, which acts as an inhibitor of the Wnt/β-catenin signaling pathway.

(4) nSC-EVs + XAV939 group: NSCs are treated with both nSC-EVs and 10 μM XAV939.

2) All groups were incubated at 37℃ with 5% CO₂.

3) After incubation, Western blotting was performed to assess NSC proliferation and changes in the Wnt/β-catenin signaling pathway.

**10 | Isolation of primary mouse spinal cord microvascular endothelial cells and collection of conditioned medium**

Neonatal mice were euthanized by cervical dislocation, disinfected in 75% ethanol for 5 min, and transferred to a biosafety cabinet. The vertebral column was exposed, longitudinally dissected, and removed; spinal cord tissue was carefully extracted with surface membranes stripped under a dissecting microscope. The tissue was minced into ~1 mm^3^ fragments, washed with PBS containing penicillin-streptomycin, and digested at 37℃ with gentle shaking for 1 h, followed by trituration until no tissue clumps remained. The pellet was subjected to a second digestion for 40 min, centrifuged at 800 × g for 5 min, and washed once. Microvascular segments were enriched by resuspension in separation medium and centrifugation at 1000 × g for 25 min; the buffy coat microvascular layer was collected, washed at 800 × g for 5 min, and resuspended in endothelial cell growth medium for culture. For conditioned medium collection, confluent endothelial cells were washed with PBS and incubated in serum-free ECM for 24 h; the supernatant was sequentially centrifuged at 500 × g (10 min) and 2000 × g (20 min) to remove debris and apoptotic bodies, sterile-filtered (0.22 μm), aliquoted, and stored at –80℃.

**11 | Recombinant Agrin and Agrin Neutralizing Antibody Treatments**

Recombinant mouse Agrin (*Agrn*) partial fragment (Arg1130–Cys1401; IPODIX Biological, IPD-X23188, *E. coli* expression, N-terminal His tag) was reconstituted in endotoxin-free ddH_2_O at 0.5 mg/mL, buffer-exchanged into PBS containing 0.1% BSA (R&D Systems, RB02), and sterilized by filtration through 0.22 μm membranes after endotoxin removal. The protein was aliquoted and stored at –80°C. Agrin neutralizing antibody (goat polyclonal IgG; R&D Systems, AF550-SP) was reconstituted in sterile PBS at 0.2 mg/mL, aliquoted, and stored at –20°C. For functional assays, DRG explants or dissociated neurons were treated with the following conditions: (1) adult spinal cord endothelial cell conditioned medium (Adult CM); (2) neonatal spinal cord endothelial cell conditioned medium (Neonatal CM); (3) Neonatal CM pre-incubated with Agrin neutralizing antibody (10 μg/mL) for 30 min at 37°C; or (4) Adult CM supplemented with recombinant Agrin (50 ng/mL).

**12 | AAV9/BBB-mediated Agrin (*Agrn*) knockdown experiment**

This study utilized AAV-mediated RNA interference to establish a specific *Agrn* gene knockdown model. The shRNA target sequence for *Agrn* was confirmed specific *via* BLAST. The double-stranded oligonucleotide sequences were as shown in Table S2.

These were cloned into the AAV vector *via* BamHI/EcoRI sites. Based on AAV9's high BBB and placental barrier penetration, and modified with a BBB-targeting peptide to enhance delivery efficiency, the viral injection protocol was optimized through preliminary experiments. Ultimately, on embryonic day 11.5 (E11.5), pregnant mice received tail vein injection of 2.5×10^11^ viral particles per mouse of recombinant AAV9/BBB-m-Agrn shRNA1-ZsGreen (experimental group) or empty vector control (control group) (*Huang et al. 2020*).

To confirm gene silencing, Agrin (*Agrn*) expression was assessed at the mRNA and protein levels in spinal cord tissue on postnatal day 7. nSC-EVs were isolated, and *in vitro* axon growth models were used to study the effects of Agrin (*Agrn*) knockdown on nSC-EVs-mediated neuronal regulation.

**13 | Reconstitution of Agrin-Deficient nSC-EVs with Recombinant Agrin via Sonication-Based Active Loading**

To reconstitute Agrin-deficient nSC-EVs with recombinant Agrin (rAgrin), we employed a sonication-based active loading protocol adapted from a previously described protocol (*Haney et al. 2019*). nSC-EVs isolated from Agrin (*Agrn*)-knockdown tissues were mixed with rAgrin (70 μg in 70 μL PBS, 1 mg/mL final concentration), subjected to bath sonication at room temperature for 3 min, and subsequently purified by nanodialysis using 30 nm polycarbonate membranes to remove unincorporated protein. The resulting rAgrin-loaded nSC-EVs were characterized by transmission electron microscopy for morphology, Western blot for canonical exosomal markers (HSP90 and TSG101), and dynamic light scattering for hydrodynamic diameter, polydispersity index, and zeta potential. Protein loading capacity and encapsulation efficiency were quantified by BCA assay, calculated as drug loading (%) = mass of loaded drug / mass of EVs × 100% and encapsulation efficiency (%) = mass of loaded drug / total mass of input drug × 100%. The functional rescue capacity of rAgrin-loaded nSC-EVs was evaluated by assessing neurite outgrowth in both DRG explant cultures and dissociated primary DRG neurons, with quantification of total neurite area and mean neurite length using ImageJ.

**14 | Cell proliferation assays**

Cell EdU Proliferation Assay: Cell proliferation of NSCs was assessed using the Cell-Light EdU Apollo567 kit. Briefly, digested NSCs were seeded at 5000 cells per well and incubated with 50 μM EdU working solution for 24 h. Following fixation, permeabilization and Apollo staining according to the manufacturer’s instructions, fluorescent images were captured, and EdU-positive cells were quantified with ImageJ.

Tissue BrdU Proliferation Assay: The tissue BrdU proliferation assay was performed according to the manufacturer’s instructions. Starting 72 h before the collection, experimental mice received intraperitoneal injections of BrdU. The BrdU solution used in the experiment was a sterile 10 mg/mL solution prepared in PBS for *in-vivo* injection. The injection volume was 100-200 μL per mouse (equivalent to 1-2 mg of BrdU), administered once every 12 h.

After the injection procedure, spinal cord tissues within 10 mm of the injury center were collected. These tissues were then subjected to immunofluorescence double-labeling experiments using mouse anti-β-III-Tubulin antibody (Abcam, ab78078, diluted at 1:200) and BrdU (Abcam, ab6326, diluted at 1:250). This experiment was carried out to observe the number of cells positive for both β-III-Tubulin and BrdU, thereby providing relevant experimental data for subsequent research analysis.

**15 | Fluorescent ubiquitination-based cell cycle indicator system for monitoring the cell cycle**

In this study, we utilized the FUCCI system to monitor the cell cycle of NSCs in real-time. The lentiviral vector was purchased from Genechem (Shanghai, China) and expresses the mKO2-hCDT1(30–120) and mAzamiGreen-hGEM(1–110) reporter modules under the EF1α promoter, separated by a T2A self-cleaving peptide, followed by a PGK-Puro cassette. This system allows specific labeling based on the ubiquitination-mediated degradation of cell cycle-dependent proteins: the hCDT1 fragment is degraded during S phase, confining red fluorescence to G1 phase; the hGEM fragment is degraded during G1 phase, confining green fluorescence to S/G2/M phases; cells in the G1/S transition exhibit yellow fluorescence due to signal overlap (*Koh et al., 2017*).

Primary-cultured NSCs were transduced with the FUCCI lentivirus to establish a stable reporter line. The infected NSCs were then treated with three types of EVs: nSC-EVs, aSC-EVs, and oSC-EVs, along with a PBS control. Finally, the high-content imaging system ImageXpress Micro (Molecular Devices) was employed to dynamically capture and quantitatively analyze the fluorescence signals, determining the cell cycle phases based on the intensity distribution of the fluorescence channels.

**16 | Preparation and testing of ACPP-modified nSC-EVs**

1) Preparation process: As per the methods reported by Shen et al (*Shen et al. 2021*), and previously described in our studies (*Yang et al. 2025*), the preparation process began with synthesizing ACPP-modified nSC-EVs by first formulating a Tris-HCl buffer. Then, preparing the DSPE solution and target peptide solution followed. Specifically, the target peptide EEEEEEEE (E8)-6-aminohexanoyl-PLGLAG-RRRRRRRR (R8) was dissolved in 1 mL of Tris-HCl buffer. The peptide solution was slowly added to the DSPE solution, and the peptide container was rinsed twice with Tris-HCl buffer; the combined rinses were added to the reaction mixture, bringing the total volume to 3.2 mL. Subsequently, 1.8 mL of Tris-HCl buffer was added to achieve a final volume of 5 mL. The reaction tube was then wrapped in aluminum foil to avoid light and mixed overnight on a shaker to promote the reaction. Next, nSC-EVs were added to the reaction system, and further incubated to complete the coupling reaction. After the reaction, the mixture was transferred into a dialysis bag and dialyzed overnight against 2 L of ultrapure water with an appropriate amount of anhydrous ethanol added, under stirring conditions. The dialysis water was replaced every 12 h to ensure thorough purification of the product. Finally, the number of purified EVs was determined by NTA for subsequent experiments.

2) Mass-spectrometric characterization: ACPP@nSC-EVs were subsequently subjected to mass-spectrometric analysis. Engineered vesicles were lysed and the proteins were digested with trypsin; the resulting peptides were purified and analyzed by liquid chromatography–tandem mass spectrometry (LC-MS/MS). Raw spectra were processed to identify signature proteins and peptides, confirming ACPP conjugation on nSC-EVs. These data establish the identity and structural integrity of the engineered ACPP@nSC-EVs and provide a validated foundation for all downstream experiments.

3) Small animal *in vivo* imaging and tracking: In this experiment, we explored the *in vivo* distribution of nSC-EVs and their targeting delivery efficacy using *in vivo* imaging techniques. First, we conjugated ACPP@nSC-EVs with the DiR dye to produce DiR-ACPP@nSC-EVs for real-time tracking of their *in vivo* dynamics. As a control, nSC-EVs were labeled with DiR alone (DiR-nSC-EVs). Next, both labeled nSC-EVs were administered to SCI mice *via* tail vein injection per mouse. After 12 hours, *in vivo* imaging was performed using an IVIS instrument. Post-imaging, major organs (heart, spleen, liver, lungs, kidneys, brain, and spinal cord) were excised for ex vivo fluorescence detection to evaluate the *in vivo* distribution and targeting efficiency of nSC-EVs. By comparing the fluorescence intensity at the SCI site between the DiR-ACPP@nSC-EVs and DiR-nSC-EVs groups, we confirmed the targeting delivery of ACPP@nSC-EVs to the injured area.

**17 | Therapeutic dosage and assessment of ACPP@nSC-EVs for SCI**

Our study used *in vivo* experiments to explore the functions and mechanisms of ACPP@nSC -EVs for SCI. The experimental design and dosage regimen of ACPP@nSC-EVs were based on previously published EVs related studies. Mice were treated *via* tail-vein injection. Experimental mice received EVs-containing PBS, while controls got the same volume of EVs-free PBS (Vehicle group), ensuring result reliability and comparability.

(1) Experimental design and grouping

This study employed a randomized controlled experimental design, with healthy female adult mice aged 8-10 weeks serving as research subjects. In our previous studies, ACPP conjugation preserved the intrinsic bioactivity of EVs, including their immunomodulatory function, while endowing them with targeted delivery to the injured spinal cord (*Yang et al. 2025*). All experimental mice were randomly and evenly allocated into three groups: the sham group, the post-SCI vehicle control group, and the intervention group (ACPP@nSC-EVs treatment group). Mice in the intervention group received tail vein injection of ACPP@nSC-EVs suspension (100 μg total EV protein per mouse in 200 μL sterile PBS). The control group was administered an equal volume of sterile PBS via the same intravenous route. The administration schedule was set at one intravenous infusion per week.

(2) Safety monitoring system

To comprehensively evaluate the safety of the experiment, a multilevel monitoring system was established throughout the experimental process: a) Baseline physiological indicators of experimental animals, including body weight, body temperature, and spontaneous activity, were monitored at fixed times daily. b) At the experimental endpoint, systemic toxicity assessment was conducted. Major organs such as the heart, liver, spleen, lungs, and kidneys were collected postmortem, and subjected to H&E Staining to observe histopathological changes. c) Liver function indicators (ALT, AST, TP) and kidney function indicators (CREA) were measured using an automatic biochemical analyzer. d) Blood compatibility was assessed by incubating venous whole-blood samples with ACPP@nSC-EV solution, Triton X-100 (positive control), or PBS (negative control) at 37 °C for 4 h.

(3) Neuromuscular and behavioral assessment

To evaluate the intervention effects, a triple behavioral validation system was established in this study. a) The BMS scoring system was employed to assess the motor function of mice in a double-blind manner at 1, 3, 7, 14, 28, 35, and 42 dpi. b) At 42 dpi, the CatWalk XT gait analysis system was used to quantitatively analyze gait-related parameters such as gait cycle, swing speed, and pressure distribution. c)Then, electrophysiological testing was conducted to measure the spinal cord nerve conduction latency and action potential amplitude.

**FIGURE S1 | Establishment of spinal cord crush injury models in neonatal, adult, and aged mice and their functional recovery outcomes.** (**A**) Representative images showing successful transverse clamp marks immediately post-injury. (**B**) Gross morphology of injured spinal cords across age groups. (**C**) Functional recovery assessed by BMS score at 42 dpi (*n* = 6); Statistical significance was determined by one-way ANOVA followed by Tukey's multiple comparisons post hoc test (C). Data are presented as mean ± SD. Significance levels are indicated as ^*^*P* < 0.05, and ^***^*P* < 0.001.

 **FIGURE S2 | Mfuzz clustering and functional annotation of spinal cord genes in neonatal and adult mice after SCI.** (**A-B**) Gene cluster expression patterns (left) and significantly enriched biological processes (right) in neonatal (A) and adult (B) mice before and after injury (1, 3 and 7 dpi).

**FIGURE S3 | Annotation and integrated analysis of snRNA-seq from neonatal, adult, and aged individuals.** (**A-B**) Volcano plot showing single-cell marker genes (A), and single-cell level distribution differences of neonatal mouse-specific highly expressed genes identified from bulk transcriptomic data (B). Statistical significance: ^***^*P* < 0.001.

**FIGURE S4 | Validation of CNS-resident Schwann-like cells in the injured spinal cord.** Representative confocal images of spinal cord sections at 3 dpi, immunostained for S100β, MBP, Olig2, and DAPI.

 **FIGURE S5 | Single-cell activity of previously identified neonatal-and aging-specific functional gene sets after SCI.** (**A-F**) Activity of neonatal-upregulated pro-neurogenic/growth gene sets across age groups. (**G-L**) Activity of aging-upregulated pro-inflammatory/cell death gene sets across ages. (**M**, **N**, **Q** and **R**) Neonatal pro-neurogenic/growth gene set activity across time points (M and N) and cell types (Q and R). (**O**, **P**, **S** and **T**) Aging pro-inflammatory/cell death gene set activity across time points (O and P) and cell types (S and T). Statistical significance: ^***^*P* < 0.001, ^****^*P* < 0.0001.

**FIGURE S6 | Analysis of differences in the quantity and activity of single-cell communication across age groups after SCI.** (**A**) Heatmap showing the count of cell-cell communication interactions between cell types; color indicates interaction number. (**B**-**D**) Age-specific communication networks in neonatal (B), adult (C), and aged (D) mice. (**E-F**) Stacked bar plots showing differential signaling weights in neonatal versus adult (E) and neonatal versus aged (F) comparisons. Rose pink: pathways elevated in neonates; lake blue/cyan-green: higher in adult/aged; black: conserved across age groups. (**G**) Functional enrichment of differential communication modules. Rose pink: functions elevated in neonates; turquoise: enriched in adult and aged groups. (**H**) Temporal activity patterns of EVs-related gene sets during SCI progression *via* AUCell scoring. Statistical significance: ^***^*P* < 0.001.

**FIGURE S7 | Isolation and characterization of EVs from primary NSCs and neurons.** (**A**) Neurosphere formation by NSCs under suspension culture conditions at day 3. (**B**) Immunofluorescence staining confirmed the expression of classic NSCs markers, Sox2 and Nestin, validating the cellular identity. (**C**) Primary mouse spinal cord neurons were isolated and identified by immunofluorescence staining for the neuronal marker β-III-Tubulin. (**D**) TEM images revealed the typical morphological features of EVs derived from both NSCs and neurons. (**E**) ExoView platform was used for further sensitive detection and characterization of EV surface markers. (**F**) NTA was used to characterize the size distribution of the diluted EVs. (**G**) Western blotting confirmed the expression of specific EV protein markers. (**H**) EV yield across cell culture and tissue sources (*n* = 8). NSCs and neurons were plated at one million cells. Mouse spinal cord tissue was dissociated and processed under the identical EV isolation protocol. EV yields are expressed per one million viable cells (cell cultures) or per 50 mg tissue equivalent. Statistical significance was determined by one-way ANOVA followed by Bonferroni post hoc test. Data are presented as mean ± SD. Significance levels are indicated as ns (not significant, *P* ≥ 0.05), and *^***^P* < 0.001.

**FIGURE S8 | Age-dependent remodeling of the SC-EVs-miRNA cargo and target gene prediction.** (**A**) PCA of miRNA expression profiles in nSC-EVs, aSC-EVs, and oSC-EVs. (**B**) Volcano plots of differentially expressed miRNAs between neonatal versus adult, old versus adult, and old versus neonatal SC-EVs (|log₂FC| ≥ 1, adjusted *P* < 0.05). (**C-D**) Venn diagrams of stringent-filtered known miRNAs (|log₂FC| ≥ 2, adjusted *P* < 0.05). (C) 85 Neo-common-down miRNAs in nSC-EVs. (D) 35 Neo-common-up miRNAs in nSC-EVs. (**E-F**) UpSet plots of target gene intersection across five databases (TargetScan, miRDB, miRanda, miRTarBase, DIANA-microT). (E) 140 target genes of Neo-common-down miRNAs. (F) 21 target genes of Neo-common-up miRNAs.

**FIGURE S9 | GO enrichment analysis of target genes regulated by age-dysregulated miRNAs in neonatal SC-EVs.** (**A**) Biological processes enriched among 140 target genes de-repressed by downregulated Neo-common-down miRNAs. (**B**) Biological processes enriched among 21 target genes suppressed by upregulated Neo-common-up miRNAs.

**FIGURE S10 | Differential protein expression and functional enrichment of EVs from different age groups.** (**A**) Number of proteins significantly up-or down-regulated in nSC-EVs and oSC-EVs compared to aSC-EVs (|logFC| ≥ 1, *P* < 0.05). (**B**-**C**) Functional enrichment analysis of differentially expressed proteins shown in modular diagrams; colored sectors represent enriched functions in up-or down-regulated protein sets. (**D**) GO functional enrichment by GSEA for nSC-EVs versus aSC-EVs. (**E**) Functional enrichment analysis *via* GSEA for oSC-EVs compared to aSC-EVs.

**FIGURE S11 | Proteomic profiling of EVs derived from neonatal, adult, and aged spinal cord tissues.** (Left) Mfuzz clustering of 7142 EVs proteins into three expression clusters (C1–C3). (Middle) Heatmap displaying expression profiles of protein clusters across developmental stages. (Right) Functional enrichment analysis of clustered proteins; bar length and label size indicate pathway significance and protein count, respectively.

**FIGURE S12 | Integrative analysis of miRNA–protein regulatory networks across age groups.** (**A**) Venn diagrams showing the intersection of target genes de-repressed by downregulated miRNAs in nSC-EVs with highly expressed proteins in nSC-EVs (3,463 proteins). Experimentally validated (miRTarBase/TarBase) and four-database-predicted targets are shown. Union and deduplication yielded 111 unique genes (red). (B) Venn diagrams showing the intersection of target genes suppressed by upregulated miRNAs in nSC-EVs with highly expressed proteins in a/oSC-EVs (3,679 proteins). Union and deduplication yielded 27 unique genes (red). (C) Radar chart comparing GO enrichment of the intersection genes. Red: Intersection of nSC-EVs de-repressed targets and highly expressed proteins. Blue: overlap between a/oSC-EVs targets and highly expressed proteins.

 **FIGURE S13 | Correlation analysis between EV proteomics and spinal cord transcriptomics after SCI.** (**A**) ssGSEA scores of nSC-EVs enriched proteins in uninjured neonatal, adult, and aged spinal cords (bulk transcriptomics) (*n* = 3). (**B** and **C**) Distribution patterns of nSC-EVs specific proteins across age groups (B) and cell types (C) in snRNA-seq datasets post-SCI. (**D**) Temporal expression dynamics of nSC-EVs protein signatures in neonatal mice after SCI. (**E** to **G**) Distribution of genes encoding nSC-EV, aSC-EV, and oSC-EV enriched proteins across spinal cord cell types before and after SCI, assessed by AUCell scoring. (**H**) Expression changes of Top20 molecules in spinal cord transcriptomes and SC-EVs proteomes across age groups. (**I**) Functional enrichment of the Top 20 molecules. Statistical significance was determined by One-way ANOVA followed by Tukey's multiple comparisons post hoc test (A). Data in A are presented as mean ± SD. Significance levels are indicated as ns (not significant, *P* ≥ 0.05), ^**^*P* < 0.01, *^***^P* < 0.001, and ^****^*P* < 0.0001.

**FIGURE S14 |** nSC-EVs are internalized by neurons (**A**) and induce neuroregenerative molecular alterations (positive regulation of neurogenesis) in NSCs (**B**).

**FIGURE S15 |** (**A**-**B**) EdU staining assessed NSC proliferation after nSC-EVs, aSC-EVs, or oSC-EVs treatment.

**FIGURE S16 | Endothelial-derived Agrin mediates neurotrophic activity.** (**A**) Agrin protein levels in nSC-EVs, aSC-EVs, and oSC-EVs (*n* = 3). (**B**) *Agrn* gene expression in neonatal and adult mice at 1 dpi, 3 dpi, and 7 dpi (*n* = 4). (**C**) AUCell scores for positive regulation of neurogenesis (GO:0050769) in *Agrn*^+^ endothelial cells across neonatal, adult, and aged mice following SCI. (**D**) AUCell scores of EV-associated gene sets in *Agrn*^+^ endothelial cells from neonatal mice after SCI. (**E**) Bright-field images of primary neonatal and adult spinal cord endothelial cells. (**F**) Immunofluorescence validation of CD31 expression in primary neonatal and adult spinal cord endothelial cells. (**G**) Quantification of CD31^+^/DAPI^+^ cell ratio in primary endothelial cell cultures, confirming comparable endothelial purity between neonatal and adult preparations (*n* = 6). (**H-I**) Representative images and quantification of DRG explant cultures stained for NF-200 (green), NeuN (red), and DAPI (blue) (*n* = 6). (**J-K**) Representative images and quantification of dissociated DRG neurons stained for β-III-Tubulin (green) (*n* = 6). Statistical significance was determined by unpaired two-tailed Student's t test (B, G) or one-way ANOVA followed by Tukey's multiple comparisons post hoc test (A, I, K). Data in A, B, G, I and K are presented as mean ± SD. Significance levels are indicated as ns (not significant), ^*^*P* < 0.05, ^***^*P* < 0.001, and ^****^*P* < 0.0001.

**FIGURE S17 | *Agrn* (Agrin) knockdown validation and characterization of rAgrin-loaded nSC-EVs.** (**A**) RT-qPCR validation of *Agrn* knockdown efficiency (*n* = 6). (**B** to **C**) Immunofluorescence staining verified Agrin protein knockdown in the spinal cords of neonatal mice (*n* = 6). (**D**) Agrin expression was significantly decreased in nSC-EVs after Agrin knockdown. (**E**) TEM images showing typical cup-shaped morphology of nSC-EVs (shNC), nSC-EVs (sh*Agrn*), and nSC-EVs (sh*Agrn* + rAgrin). (**F**) Western blot analysis of EV markers HSP90 and TSG101 confirming preserved marker expression in all three groups. (**G-H**) Zeta potential (left) and polydispersity index (PDI, right) measurements. Zeta potential shifted from -41.70 mV to -29.86 mV upon rAgrin loading (*P* < 0.05), while PDI remained unchanged (ns, not significant). Statistical significance was determined by two-tailed Student’s t test. Significance levels are indicated as ns (not significant), ^*^*P* < 0.05, ^**^*P* < 0.01.

**FIGURE S18 | Enhanced targeted delivery of ACPP-modified nSC-EVs to the SCI site.** (**A**) Schematic of ACPP conjugation onto nSC-EVs. (**B**) Representative DIA mass spectrum of the ACPP peptide on engineered nSC-EVs. Fragment ions (red, y ions; blue, b ions) match the E8-PLGLAGR region of the ACPP sequence (EEEEEEEE-6-aminohexanoyl-PLGLAG-RRRRRRRR); the 6-aminohexanoyl linker and poly-Arg tail are not covered by de novo sequencing. Error plots show mass accuracy within ±0.05 Da. (**C**) Molecular docking demonstrating specific binding between ACPP and MMP2/MMP9, with hydrogen bonds indicated. (**D-G**) Ex vivo imaging revealed significantly higher fluorescence signals in CNS tissues (brain and spinal cord) after ACPP@nSC-EVs injection compared to controls (*n* = 6). (**H**) Representative images show fluorescence signals in the liver and spinal cord (*n* = 6). (**I**) Representative quantitation of fluorescence signals in the ex vivo liver (*n* = 6). Statistical significance was determined by two-tailed Student’s t test (F, G, I). Data are presented as mean ± SD. Significance levels are indicated as ns (not significant), ^*^*P* < 0.05, ^**^*P* < 0.01.

**FIGURE S19 | *In vivo* safety evaluation of ACPP@nSC-EVs.** (**A**) Histological analysis of major organs after *in vivo* administration of ACPP@nSC-EVs (H&E staining). (**B** to **E**) Effects of ACPP@nSC-EVs on biochemical markers including ALT, AST, TP, and CREA (*n* = 6). (**F**) Assessment of hemolysis following ACPP@nSC-EVs administration (*n* = 6). Statistical significance was determined by two-tailed Student’s t test (B–E) or one-way ANOVA followed by Tukey’s post hoc test (F). Data in B–F are presented as mean ± SD. Significance levels are indicated as ns (not significant) and ^****^*P* < 0.0001.
**Table S1.**

Primer sequences used for RT-qPCR analyses in this study.

| Gene | Sequence (forward 5’-3’) | Sequence (reverse 5’-3’) |
| --- | --- | --- |
| *Agrn* | CTGGCCAATGAGATCCCCG | AATGGCCAGTGCCACATAGT |
| *GAPDH* | CATCACTGCCACCCAGAAGACTG | ATGCCAGTGAGCTTCCCGTTCAG |

**Table S2.**

The double-stranded oligonucleotide sequences for *Agrn* were as follows.

| *Agrn* | Sequence (5’-3’) |
| --- | --- |
| Top strand | GATCCGCTCAGATGCTTCCACCTATACTCGAGTATAGGTGGAAGCATCTGAGCTTTTTTG |
| Bottom strand | AATTCAAAAAAGCTCAGATGCTTCCACCTATACTCGAGTATAGGTGGAAGCATCTGAGCG |

**Table S3.**

Characterization of nSC-EVs (sh*Agrn*) before and after rAgrin loading

| **Parameter** | **Before loading nSC-EVs (sh*Agrn*)** | **After loading nSC-EVs (sh*Agrn*+rAgrin)** |
| --- | --- | --- |
| Morphology (TEM) | Typical cup-shaped structure | Typical cup-shaped structure |
| EVs markers (WB) | HSP90 (+), TSG101 (+) | HSP90 (+), TSG101 (+) |
| Hydrodynamic size (nm) | 162.03 ± 6.41 | 180.10 ± 3.71 |
| PDI | 0.244 ± 0.007 | 0.246 ± 0.025 |
| Zeta potential (mV) | −41.70 ± 2.80 | −29.86 ± 3.69 |
| Cargo loading (%) | — | 3.35 |
| Encapsulation efficiency (%) | — | 25.35 |

*Drug loading and encapsulation efficiency were calculated using the following formulas: Drug loading (%) = mass of loaded drug / mass of EVs × 100%; Encapsulation efficiency (%) = mass of loaded drug / total mass of input drug × 100%; Data are presented as mean ± standard deviation (n = 3). PDI, polydispersity index; rAgrin, recombinant Agrin.*

**Data S1. (separate file)**

Cluster-specific spinal cord gene names in neonatal, adult, and aged mice.

**Data S2. (separate file)**

Temporal dynamics of high-activity gene sets in three age stage were compared between neonatal and adult mouse spinal cords before and after injury (1, 3 and 7 dpi).

**Data S3. (separate file)**

Single-cell marker gene expression level.

**Data S4. (separate file)**

Clustering analysis of protein expression levels in spinal cord-derived EVs from neonatal, adult, and aged mice.

**Data S5. (separate file)**

Expression levels of neonatal upregulated conserved molecules in tissue transcriptomes and EV proteomes.
